# Inter-individual variability amplified through breeding reveals control of reward-related action strategies by Melanocortin-4 Receptor in the dorsomedial striatum

**DOI:** 10.1038/s42003-022-03043-2

**Published:** 2022-02-08

**Authors:** Aylet T. Allen, Elizabeth C. Heaton, Lauren P. Shapiro, Laura M. Butkovich, Sophie T. Yount, Rachel A. Davies, Dan C. Li, Andrew M. Swanson, Shannon L. Gourley

**Affiliations:** 1 Department of Pediatrics and Children’s Healthcare of Atlanta, Emory School of Medicine, Atlanta, GA USA; 2 Yerkes National Primate Research Center, Emory University, Atlanta, GA USA; 3 Graduate Program in Neuroscience, Emory University, Atlanta, GA USA; 4 Graduate Program in Molecular and Systems Pharmacology, Emory University, Atlanta, GA USA

**Keywords:** Reward, Motivation

## Abstract

In day-to-day life, we often must choose between pursuing familiar behaviors or adjusting behaviors when new strategies might be more fruitful. The dorsomedial striatum (DMS) is indispensable for arbitrating between old and new action strategies. To uncover molecular mechanisms, we trained mice to generate nose poke responses for food, then uncoupled the predictive relationship between one action and its outcome. We then bred the mice that failed to rapidly modify responding. This breeding created offspring with the same tendencies, failing to inhibit behaviors that were not reinforced. These mice had less post-synaptic density protein 95 in the DMS. Also, densities of the melanocortin-4 receptor (MC4R), a high-affinity receptor for α-melanocyte-stimulating hormone, predicted individuals’ response strategies. Specifically, high MC4R levels were associated with poor response inhibition. We next found that *reducing Mc4r* in the DMS in otherwise typical mice expedited response inhibition, allowing mice to modify behavior when rewards were unavailable or lost value. This process required inputs from the orbitofrontal cortex, a brain region canonically associated with response strategy switching. Thus, MC4R in the DMS appears to propel reward-seeking behavior, even when it is not fruitful, while moderating MC4R presence increases the capacity of mice to inhibit such behaviors.

## Abstract (web summary)

To uncover mechanisms underlying reward-seeking behaviours, Allen et al. employ a task in which mice must arbitrate between rewarded and nonrewarded behaviors. They demonstrate that individual differences in response flexibility are associated with melanocortin-4 receptor density in the dorsomedial striatum.

## Introduction

In day-to-day life, we often pursue familiar behavioral sequences that have been reinforced in the past – e.g., driving a familiar route home from work – or inhibit behaviors when they fail to be reinforced – like avoiding that route when construction blocks our path. The dorsomedial, or associative, striatum (DMS), roughly analogous to the primate caudate, is indispensable for arbitrating between familiar and new action strategies. For instance, damage to the DMS causes rats to pursue familiar behavioral sequences even when they cease to be rewarded^1–5^. Motor task learning recruits neural ensembles in the DMS that decline in activity with task proficiency^6^. Further, instrumental conditioning – learning to perform a behavior for reward – triggers immediate-early gene expression and transcriptional activity in the DMS^7–10^ and requires direct spiny projection neurons in the DMS^10^. Nevertheless, the molecular mechanisms by which the DMS coordinates the flexible modification of behavior are still emerging.

A strategy by which to identify molecular factors regulating a given behavior is to manipulate the levels or activities of proteins that are predicted to control that behavior. A limitation of this approach is that unpredicted factors – those that we might not anticipate – remain obscure. Here, we instead used a discovery-driven strategy. We first bred mice that displayed a particular behavioral trait – resistance to inhibiting behaviors when they failed to be rewarded. Their offspring displayed the same behavioral patterns, providing a tool to investigate mechanistic factors. We measured proteins associated with synaptic presence and function, these efforts ultimately leading us to the hypothesis that melanocortin-4 receptor (MC4R) in the DMS controls response flexibility – defined here as the ability to inhibit instrumental behaviors when they are not fruitful.

Melanocortins are peptide hormones including adrenocorticotropic and melanocyte-stimulating hormones. Of the five melanocortin receptors, two are primarily expressed in the central nervous system – MC3R and MC4R. MC4R is a high-affinity receptor for α-melanocyte-stimulating hormone (α-MSH) and has been intensively studied in the hypothalamus, where its role in energy homeostasis is now well-understood^11,12^. Striatal MC4R function has also been investigated for >4 decades, but overwhelmingly focused on the ventral striatum. For instance, melanocortins trigger excessive grooming^13^, which is attributable to activity at MC4R in the ventral striatum (reviewed^14^). Further, cocaine increases *Mc4r* and synaptic MC4R content in the ventral striatum, where its activity masks the aversive properties of cocaine, and also potentiates drug seeking, sensitization, cocaine-elicited grooming, and compulsive-like behaviors^14–17^.

Despite this historical focus on ventral striatal melanocortin function, dorsal striatal levels of MC4R are rich^18–20^, and their function remains incompletely understood. We found that MC4R in the DMS propels reward-seeking behavior. Meanwhile, moderating MC4R presence via site-selective gene silencing increased the capacity of mice to inhibit nonreinforced responses; this occurs at least in part via interactions with the orbitofrontal cortex (OFC), a cortical brain region canonically involved in modifying action strategies.

## Results

### Individual differences in reward-related response strategies in mice

**Fig. 1:**
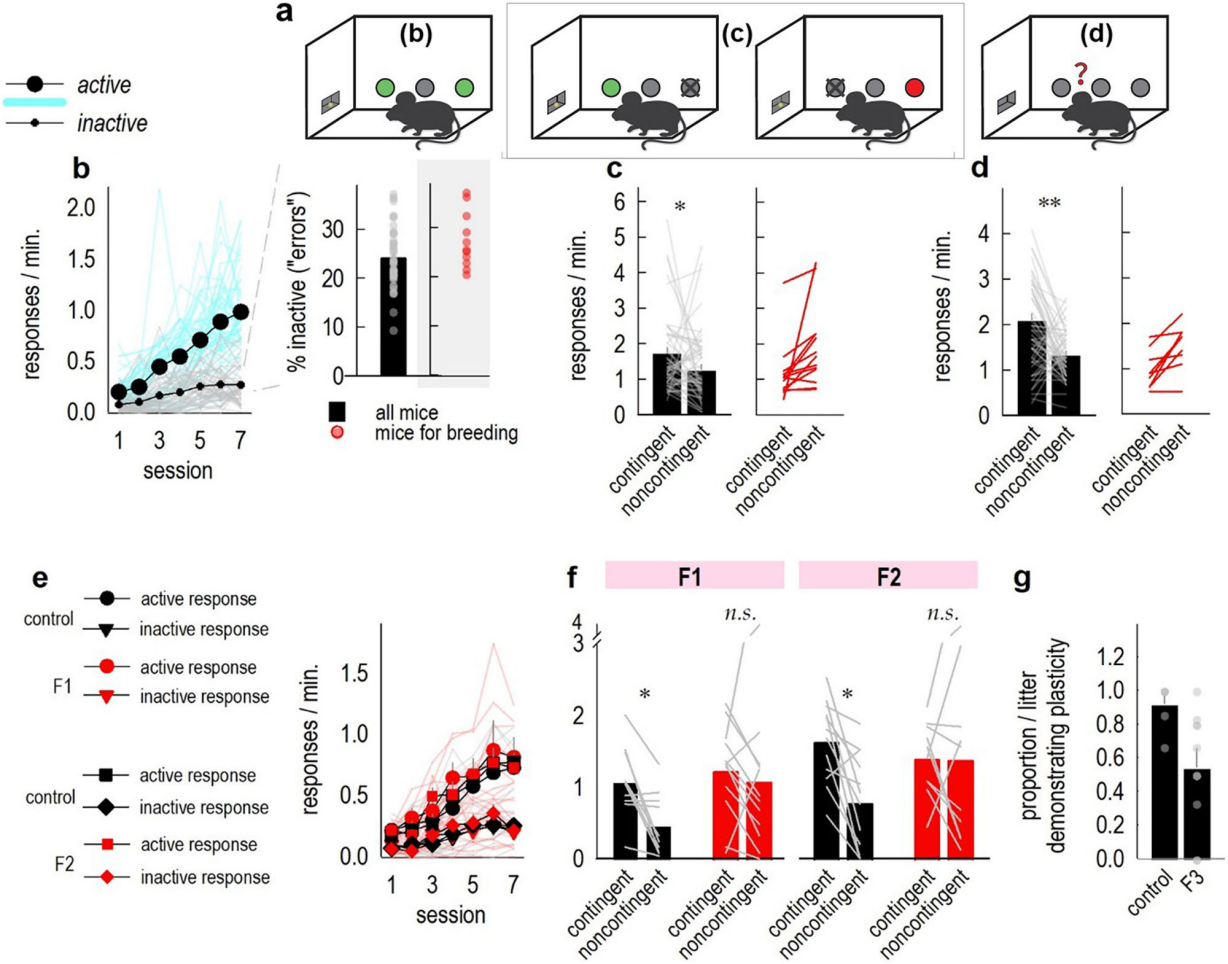
Multi-generational biases in reward-related response strategies. **a** Task schematic. Responding at two ports resulted in food pellet delivery. Next, we provided pellets associated with one response noncontingently and responding was not reinforced, while responding at the other port remained reinforced. Finally, response preference was assessed during a probe test (right). Small letters in the boxes correspond with the figures below. **b** In an initial screen of 52 mice, all mice acquired the reinforced (“active”) nose poke responses, relative to responding that was not reinforced (“inactive”) [active × day *F*_(6,306)_ = 61.95, *p* < 0.001]. Light blue and gray lines represent individual mice. Inset: Inactive responses as a percentage of all responses. Mice selected for breeding based on this criterion are shown in individual points at right (*n* = 12 selected). **c** Next, one response was no longer reinforced, and food pellets were delivered noncontingently. Mice as a group generated lower response rates during this session, relative to a session when the other response remained reinforced [paired *t*_51_ = 2.2, *p* = 0.04]. Light gray lines represent individual mice. Some of these individual mice did not follow the overall pattern; those selected for breeding are shown at right (*n* = 9 selected). **d** During a probe test, mice as a group again generated higher response rates on the port associated with reinforcement [paired *t*_51_ = 4.04, *p* < 0.001]. Light gray lines represent individual mice, and again, not all individuals followed this pattern; those selected for breeding are shown at right (*n* = 14 selected). Mice deviating from the typical pattern of responding on 2/3 measures were bred. **e** The F1 and F2 generations acquired reinforced nose poke responses [main effect of day *F*_(6,108)_ =14.4, *p* < 0.001; no main effects of group, generation, or interactions *F*s ≤ 1; no day × port × group ×  generation *F*_(6,108)_ = 2.02, *p* = 0.07]. A port × day interaction indicated that mice differentiated between the active and inactive ports with time [*F*_(6,108)_ = 30.2, *p* < 0.001; main effect of port *F*_(1,18)_ = 129.7, *p* < 0.001]. Light lines represent individual mice. **f** When responding was not reinforced and pellets were delivered noncontingently, experimental offspring as a group did not modify their behavior relative to a session when responding was reinforced [interaction *F*_(1,18)_ = 4.5, *p* = 0.048; main effect of contingency *F*_(1,18)_ = 6.6, *p* = 0.02]. We detected no effects of generation [no main effect *F*_(1,18)_ = 2.5, *p* = 0.13; no generation × contingency or generation × group interaction *F*s < 1], suggesting that the behavioral phenotype was stable. Gray line represent individual mice (*n* = 2/litter, with control F1 litter *n* = 5, F1 litter *n* = 6, control F2 litter *n* = 6, F2 litter *n* = 5). **g** Finally, in the F3 generatio*n*, we tested all possible progeny and calculated the proportion of each litter that inhibited responding when it was not explicitly reinforced. The majority of control mice displayed this response inhibition capacity, while only roughly half of experimental offspring did [Welch’s *t*_12.74_ = 3.07, *p* = 0.009]. Control F3 litter *n* = 6, F3 litter *n* = 10. Individual symbols represent individual litters. Throughout, bars and connected symbols represent means (+SEMs). *p < 0.05, **p < 0.001. *n.s*. is non-significant. F1, F2, and F3 refer to filial 1, 2, 3 generations. Illustration by author Aylet T. Allen.

Here we bred mice that displayed particular behavioral traits, with the ultimate goal of creating a tool by which to identify molecular factors controlling animals’ propensity to inhibit behaviors that are unlikely to be reinforced with desired outcomes. Fifty-two mice were initially screened. Testing occurred in three stages: *training*, when mice were trained in operant conditioning chambers to respond on two nose poke ports for food. A third, “inactive” port was never reinforced. Next occurred *noncontingent pellet delivery*, when pellets associated with one familiar response were delivered regardless of the animals’ behaviors (and responding was not reinforced); and then a brief *probe test* the next day, conducted in extinction, when mice could choose between the intact vs. now-defunct contingencies (Fig. 1a). The mice selected for breeding fulfilled two or three of the following criteria: (1) >20% of responses were directed to the inactive nose poke port during training; (2) they failed to reduce responding when pellets were delivered noncontingently (meaning, they generated the same or more responses relative to a session when pellets were delivered contingently); or (3) they failed to prefer the reinforced behavior during the probe test (meaning, they generated the same or more responses on the aperture associated with noncontingent vs. contingent pellet delivery).

In this and all other experiments, mice did not develop side biases during training that could impact later response patterns; thus, response rates on both active nose poke ports are collapsed for simplicity. Means and SEMs of all 52 mice are represented in black in Fig. 1b–d, with the individual mice that were bred in symbols at right. Mice could differentiate between active and inactive nose pokes ports during training (Fig. 1b). The inset in Fig. 1b represents total responses on the inactive port over the entire course of training. Individual points represent mice that generated >20% of all responses on the inactive port and also fulfilled another breeding criterion and thus were bred. The mice selected for breeding were not ultimately distinguishable based on this singular criterion. Thus, it seems unlikely that this behavioral characteristic contributed to later response patterns; it is included merely for transparency.

Next, one response ceased to be reinforced, and pellets associated with that response were provided noncontingently. As a group, mice inhibited responding (Fig. 1c); however, not all individuals inhibited the nonreinforced response. Those mice selected for breeding based on this criterion are represented by individual lines, highlighting their marked divergence from the group means. Similarly, in a subsequent probe test, mice as a group preferred the response associated with reinforcement (Fig. 1d), but again, some individual mice failed to demonstrate this preference. The mice selected for breeding based on this criterion are represented by individual lines, again highlighting their divergence from the group mean.

Ultimately, 15 mice were selected for breeding, and they generated 6 litters (the F1 generation), which were trained and tested identically, as were their offspring (F2). They were compared to same-age control counterparts (mice of the same strain bred in the laboratory) whose parents had also undergone identical testing. Two mice from each litter were tested, and each litter was considered a single, independent sample (the mean of mice in that litter).

Response rates during training of filial generations did not differ between groups or generations (Fig. 1e). Next, one port was occluded, and responses on the remaining port ceased to be reinforced; instead, pellets were delivered noncontingently. Control mice overwhelmingly inhibited responding during this session, relative to a session when the other port was available and responding was reinforced. Meanwhile, response patterns in the experimentally bred mice were less flexible, as can be appreciated in Fig. 1f. As an additional example of this phenomenon: response rates in the control mice in Fig. 1f were 4.1-fold higher, on average, when responding was explicitly reinforced than when it was not. Meanwhile, experimental offspring in Fig. 1f responded only twice as much on average when responding was reinforced, and they were sufficiently variable such that the contingent vs. noncontingent conditions did not statistically differ (Fig. 1f).

Interestingly, experimental offspring consistently favored the reinforced behavior during probe tests conducted a day later – like typical mice (Suppl. Fig. 1). Therefore, our breeding strategy spared contingency memory formation. Our studies thus focus on striatal factors controlling rapid, “in-the-moment” response inhibition, occurring when mice first encounter violated response-reward contingencies.

Next, we tested all progeny of the F3 generation (78 mice) and calculated the proportion of each litter that inhibited nonreinforced responses. The majority of typical offspring inhibited nonreinforced behaviors, as expected, but only about half of animals in each experimental litter inhibited responding when it was not reinforced (Fig. 1g).

We imagine that experimental offspring are slow to detect changes in response-reward links, or have difficulty inhibiting a behavioral sequence once it has been initiated. Another possibility is that they developed an impulsive-like quality, the “inability to wait”^21^, which can be tested using a delay discounting procedure. Briefly, mice are trained to respond for large and small reinforcers. When delays are introduced between responses and large reinforcers, mice shift preference from large to small reinforcer, which can be quantified. Responding during time-out periods can also be measured. Males responded more during time-out periods when they experienced long delays, as reported previously^22,23^, but we found no group differences on any measure (Suppl. Fig. 2).

### Individual differences in instrumental response strategies are associated with striatal protein composition

**Fig. 2:**
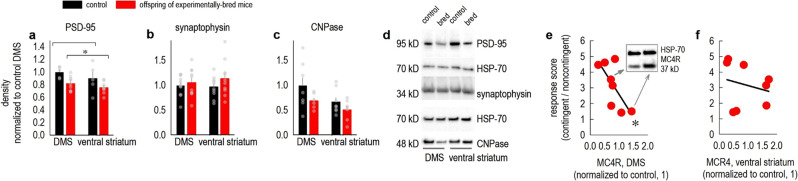
Individual differences in response flexibility associate with striatal protein content. **a** PSD-95 was measured in the dorsomedial and ventral striatum, revealing lower levels in the offspring of experimentally bred mice [main effect of group *F*_(1,23)_ = 9.6, *p* = 0.005; no effect of region *F*_(1,23)_ = 2.3, *p* = 0.14; no interaction *F* < 1]. *n* = 5–9/group. **b** Meanwhile, levels of the presynaptic protein synaptophysin did not differ between groups or regions [group *F*_(1,37)_ = 1.7, *p* = 0.2; other *F*s < 1]. *n* = 10–11/group. **c** The oligodendrocyte marker CNPase *appeared* lower in the offspring of experimentally bred mice [effect of group *F*_(1,24)_ = 4.1, *p* = 0.054; effect of brain region *F*_(1,24)_ = 4.9, *p* = 0.04; no interaction *F* < 1], but the effect was not significant. *n* = 6–8/group. **d** Representative blots loaded in the order indicated, including corresponding HSP-70 loading controls. **e** MC4R levels correlated with response scores, such that higher MC4R was associated with response inflexibility (scores ~1), and lower levels were associated with response plasticity (scores > 1) [*r* = 0.71, *p* = 0.047]. Representative blots are inset, with arrows linking each lane to the respective mouse. **f** MC4R in the ventral striatum of the same mice did not correlate with response strategies [*r* = 0.23, *p* = 0.59]. *n* = 8. Bars represent means + SEMs. Symbols represent individual mice. *p < 0.05. DMS refers to the dorsomedial striatum. All gels were run at least twice, with concordant results. Samples were normalized to the control sample mean on the same gel to control for variance across gels.

Instrumental response flexibility requires synaptic signaling in the DMS (see Introduction). Thus, we next quantified PSD-95, synaptophysin, and CNPase in the DMS and ventral striatum, for comparison. These proteins are commonly considered markers of the excitatory postsynaptic compartment, the presynaptic compartment, and mature oligodendrocytes, respectively. PSD-95 was lower in mice with poor response flexibility across both regions (Fig. 2a), while synaptophysin was unaffected (Fig. 2b). CNPase was qualitatively lower in mice with poor response flexibility (Fig. 2c, d), but this comparison did not reach significance following Benjamini–Hochberg correction for multiple comparisons.

One additional protein, MC4R, was measured based on the results of an exploratory transcriptomic analysis of the DMS from the F3 generation. MC4R levels did not differ between groups (all *p*s > 0.2, not shown). Interestingly, however, protein levels correlated with behavioral response strategies: Specifically, we distilled response strategies down to a single value by dividing response rates generated during the contingent pellet delivery/noncontingent pellet delivery sessions. Scores > 1 indicate that response rates were higher when responding was explicitly reinforced than when it was not, while scores ⁓1 indicate that mice responded equivalently in both conditions. MC4R levels negatively correlated with response ratios (Fig. 2e), suggesting that mice with high MC4R fail to inhibit responding that is not reinforced, while mice with low MC4R modify response strategies. Meanwhile, ventral striatal MC4R did not correlate with response patterns (Fig. 2f). Notably, other proteins that were predicted to co-vary with behavioral measures (α-tubulin, calmodulin, GluN2B, Tau, and tyrosine hydroxylase) ultimately did not (Suppl. Fig. 3), suggesting that striatal protein content was not grossly altered in our experimental offspring.

### MC4R control of action strategies

**Fig. 3:**
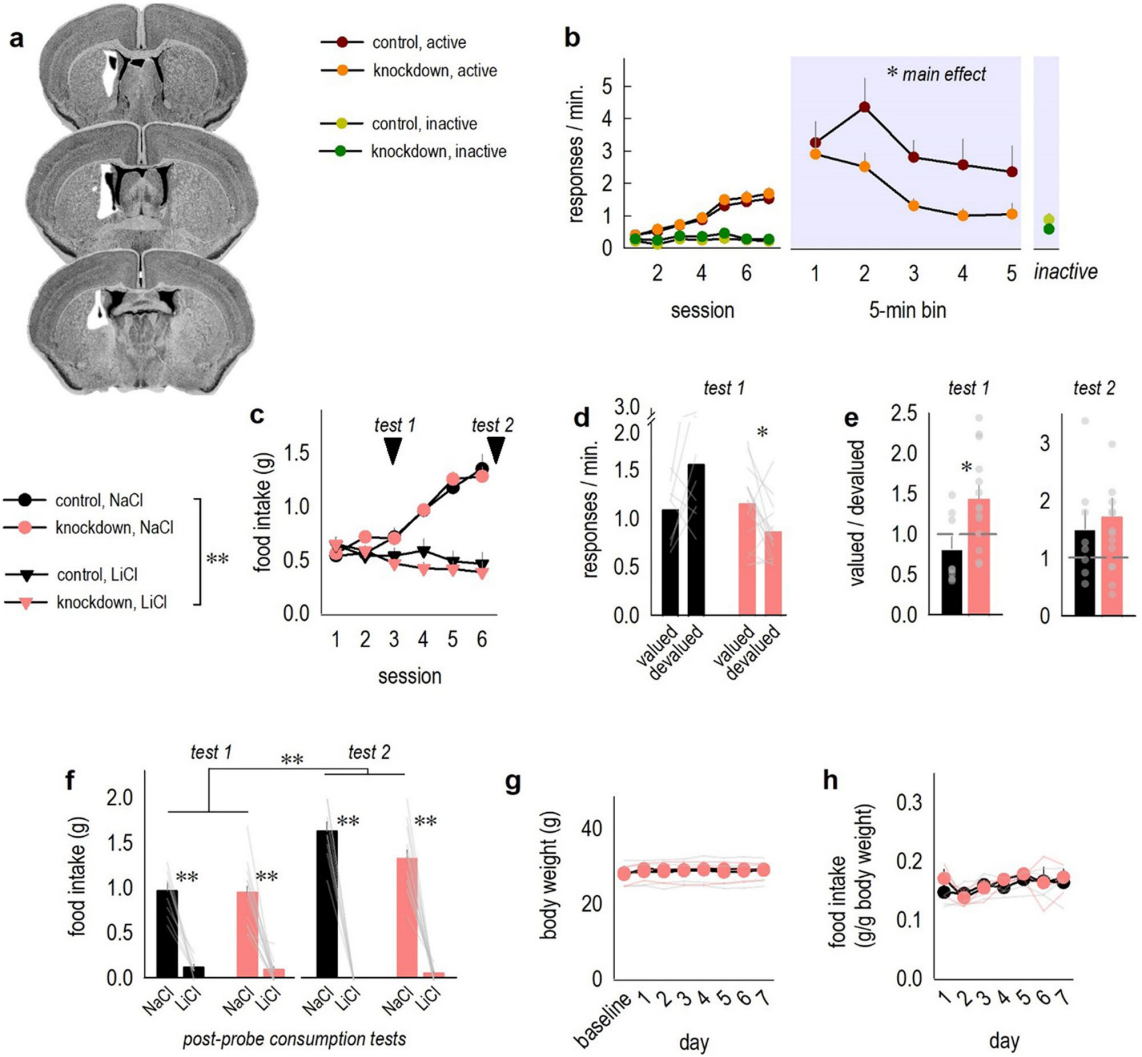
*Mc4r* knockdown in the DMS expedites response inhibition. **a** Viral vector infusion sites on coronal sections from the Mouse Brain Library^60^ represent areas of *Mc4r* knockdown. White represents large spread, with black the smallest. **b** Response acquisition (left) was unaffected, despite *Mc4r* knockdown in the DMS [no main effect of group *F*_(1,23)_ = 1.6, *p* = 0.2; main effect of day *F*_(6,138)_ = 24.8, *p* < 0.001; no group × day interaction *F* < 1]. Groups differentiated between active and inactive ports, as expected [day × port interaction *F*_(6,138)_ = 47.7, *p* < 0.001; no day × port × group interaction *F* < 1]. When pellets were delivered noncontingently and responding was not reinforced (right), responding decreased across time [main effect of time bin *F*_(4,92)_ = 6.7, *p* < 0.001], as expected, but knockdown mice res*p*onded less overall [main effect of group *F*_(1,23)_ = 4.3, *p* = 0.05; no time × group interaction, *F* < 1]. Rates on the inactive port did not differ and are collapsed for simplicity. *n* = 13 control, 12 knockdown. **c** Next, one of the reinforcer pellets was paired with LiCl (decreasing its value), while the other pellet was paired with NaCl (a control). Ad libitum consumption of the LiCl-paired pellet was far lower than the NaCl-paired pellet across repeated pairings [effect of day *F*_(5,100)_ = 21.5, *p* < 0.001; effect of pellet *F*_(1,20)_ = 49.3, *p* < 0.001; pellet × day interaction *F*_(5,100)_ = 38.5, *p* < 0.001; no pellet × group interaction *F*_(5,100)_ = 1.96, *p* = 0.09 or other effects *F*s ≤ 1]. **d** Mice were returned to the testing chambers. Early in conditioning (“test 1”), only *Mc4r* knockdown mice inhibited responding for the devalued reinforcer [interaction *F*_(1,20)_ = 9.4, *p* = 0.006; no main effect of value *F* < 1; no main effect of group *F*_(1,20)_ = 3.7, *p* = 0.07]. Light lines represent individual mice. **e** The same data were converted to preference scores (valued/devalued), in which case, scores >1 reflect response preference. The dashed line at 1 represents no change in behavior based on outcome value. Knockdown mice generated higher scores in the initial test, again indicating that they inhibited one behavior over another [*t*_20_ = 2.6, *p* = 0.02]. Following more conditioning (“test 2”), both groups inhibited responding for the devalued pellet, as expected, and did not differ [*t*_20_ = 0.4, *p* = 0.67]. **f** In post-probe consumption tests, mice overwhelmingly preferred the NaCl-paired pellet and avoided the LiCl-paired pellet, an effect that intensified with time, indicating that the CTA procedure was successful [main effect of pellet *F*_(1,20)_ = 248.6, *p* < 0.001; main effect of test 1 vs. 2 *F*_(1,20)_ = 38.4, *p* < 0.001; pellet × test interaction *F*_(1,20)_ = 72.6, *p* < 0.001]. No main effect of grou*p* was detected [*F*_(1,20)_ = 2.01, *p* = 0.17]. Light lines represent individual mice. *n* = 8 control, 14 knockdown. **g** *Mc4r* knockdown did not affect free-feeding body weights [*F*s < 1] or **h** chow intake [*F*s < 1]. Light lines represent individual mice. *n* = 4 control, 3 knockdown. Bars and connected symbols represent means + SEMs. *p ≤ 0.05, **p < 0.001. Instrumental conditioning experiments were conducted twice, with concordant results. Versio*n*s of **b** and **c** with individual mice represented are provided in Suppl. Fig. 6a, b.

Our findings predict that inhibiting MC4R presence might facilitate response inhibition. To test this hypothesis, we obtained ‘floxed’ *Mc4r* mice, a well-established tool in MC4R research, in which the single coding exon is flanked by loxP sites, and the introduction of Cre-recombinase (Cre) obstructs MC4R production^24^. Cre was delivered selectively to the DMS via CaMKII-driven adeno-associated viral vectors (Fig. 3a). *Mc4r* status did not affect response rates during training (Fig. 3b), important given that global knockout can reduce operant response rates for food^25^, and suggesting that gross locomotor activity did not differ between groups.

Next, one nose poke behavior failed to be reinforced, and instead, pellets were delivered noncontingently. We extracted response rates in bins to compare groups across time. Response rates increased as animals first experienced the contingency violation, resembling a so-called “extinction burst,” as previously reported in mice performing the same task^26^. All mice ultimately inhibited responding with time, though, importantly with *Mc4r* knockdown mice responding less overall (Fig. 3b).

To further solidify our interpretation that site-selective *Mc4r* knockdown facilitates response inhibition, we reinstated responding in *Mc4r*-deficient mice, then tested their behavioral sensitivity to reinforcer devaluation. In this case, mice will inhibit responding for a devalued outcome. Mice were given free access to one of the two reinforcer pellets in a clean cage, followed by an injection of LiCl, inducing transient malaise and decreasing the value of that pellet via conditioned taste aversion (CTA). The other pellet was paired with NaCl. With repeated pairings, typical mice will inhibit the behavior that leads to the LiCl-paired, devalued outcome, while responding for the NaCl-paired pellet will remain intact – reflecting response plasticity based on reward value [for discussion of reinforcer devaluation, see^27^. We hypothesized that *Mc4r* knockdown mice would more readily inhibit responding than control mice. To generate the resolution to detect such an effect, we tested response strategies at two time points: after only a few LiCl pairings, before pellet aversion was strong, and following more pairings, when it was robust (arrows, Fig. 3c). We envisioned that this approach might allow for the resolution to detect *enhancements* in response inhibition, if they existed.

Upon CTA, mice decreased *ad libitum* consumption of the LiCl-associated pellet, but not NaCl-paired pellet, as expected (Fig. 3c). When returned to the conditioning chambers at the early time point, control mice showed no evidence yet of changing response strategies, indicated by equivalent responding on the ports associated with the valued *vs*. devalued outcomes. Meanwhile, a majority of knockdown mice (73%) favored the response associated with the valued outcome (Fig. 3d). Thus, knockdown enriched response plasticity, triggering mice to inhibit a behavior associated with devalued food.

Group differences can be further appreciated by converting response rates to ratios: valued/devalued. Scores >1 reflect preference for the port associated with the valued pellet and neglect of the devalued pellet, while scores of ⁓1 indicate no change in behavior based on outcome value. As expected, knockdown mice generated higher ratios early in conditioning, while control mice required more CTA to generate response preferences (Fig. 3e). Thus, reducing striatal *Mc4r* expedites the ability of mice to inhibit actions when appropriate.

Importantly, following both probe tests, we assessed the propensity of mice to consume freely-available pellets placed in their cages. At both time points, both groups consumed far more of the pellet that had been paired with NaCl, relative to the pellet that had been paired with LiCl (Fig. 3f). Thus, instrumental response strategies could not be attributable to differences in CTA.

Given that hypothalamic *Mc4r* controls feeding, and our tasks are food-reinforced, it was also important to measure general food intake following DMS-specific knockdown. *Ad libitum* chow intake and body weights did not differ between groups (Fig. 3g, h).

### MC4R control of action strategies via the OFC

MC4R presence controls the localization of GluA2-containing AMPA receptors (AMPARs) at the cell membrane of striatal medium spiny neurons (MSNs). Specifically, MC4R binding triggers the internalization of these receptors^28^, leading to the hypothesis that MC4R presence may control response strategies by gating sensitivity to excitatory inputs. Implicit in this model is that behavioral effects of *Mc4r* silencing are dependent on glutamatergic afferents to the DMS.

**Fig. 4:**
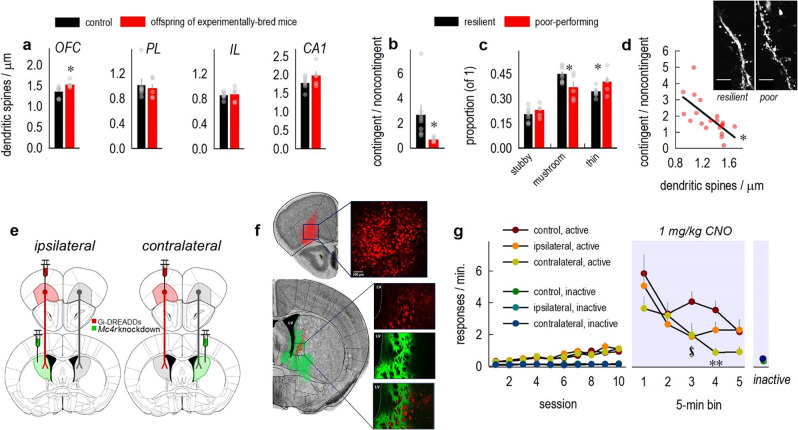
Reducing *Mc4r* in the DMS expedites response inhibition in an OFC-dependent manner. **a** Terminal dendrites on neurons in multiple brain regions from the progeny of experimentally bred mice were imaged, and dendritic spines were enumerated. Densities differed relative to typical mice in the ventrolateral OFC [*t*_11_ = 2.7, *p* = 0.02]. Meanwhile, groups did not differ in the prelimbic cortex [PL; *t*_10_ = 0.4, *p* = 0.68] or infralimbic cortex [IL; *t*_10_ = -0.2, *p* = 0.85] or hippocampal CA1 [*t*_10_ = −1.4, *p* = 0.18]. *n* = 7 control, 5–6 offspring of experimentally bred mice. **b** OFC neurons from a separate cohort of experimental progeny were next imaged. Poor-performing progeny (those generating preference scores of ∼1) were compared to resilient progeny (preference scores >1, reflecting increased responding in the contingent condition; Mann-Whitney *p* = 0.002). **c** Immature, thin-type dendritic spines on OFC dendrites were in excess in poor-performing mice, while resilient mice had more mature, mushroom-shaped spines [group × spine type interaction *F*_(2,22)_ = 4.2, *p* = 0.03; main effect of spine type *F*_(2,22)_ = 33.4, *p* < 0.001; no main effect of group *F* < 1]. *n* = 8 resilient, 5 poor-performing. **d** Further, response scores correlated with thin-type dendritic spine densities in 2 independent cohorts (*r*^2^ = 0.5, *p* < 0.001). *n* = 20. Representative dendrites at right. Scale bar=5 µm. **e** We next used an asymmetric infusion design to determine whether OFC-to-DMS projections are necessary for the response inhibition capacity conferred by *Mc4r* silencing. In the ipsilateral condition, one DMS lacks *Mc4r* (green), and the upstream OFC has Gi-coupled DREADDs (red). In the contralateral (“asymmetric”) condition, mice also experience OFC inactivation, but the healthy OFC projects to an *Mc4r* knockdown DMS. Control mice bear control viral vectors, and thus have intact MC4R levels and no DREADDs. Cartoon adapted from^59^. **f** OFC terminals overlapped with transduced regions of the DMS. Histological traces are represented on images from the Mouse Brain Library^60^. The Cre groups (i.e., *Mc4r* knockdown) are further illustrated in Suppl. Fig. 4. Scale bar=100 µm. “LV” refers to the lateral ventricle. **g** Response acquisition did not differ between groups (left) [main effect of day *F*_(9,333)_ = 24.5, *p* < 0.001; no main effect of group *F* < 1; no grou*p* × day interaction *F*_(18,333)_ = 1.03, *p* = 0.43]. Groups differentiated between active and inactive ports during training [day × port interaction *F*_(9,333)_ = 22.02, *p* < 0.001; no day × port × group interaction *F*_(18,333)_ = 1.3, *p* = 0.17]. CNO was delivered to all mice when a familiar behavior failed to be reinforced (right). The contralateral group inhibited responding more strongly than the control group [group × time interaction *F*_(8,148)_ = 2.2, *p* = 0.04; main effect of time bin *F*_(4,148)_ = 15.0, *p* < 0.001; no main effect of grou*p F*_(2,37)_ = 2.9, *p* = 0.07]. *n* = 15 control, 12 ipsilateral, 13 contralateral. Bars and connected symbols represent mea*n*s + SEMs. Other symbols represent individual mice. *p < 0.05. ^$^*p* < 0.05 contralateral and ipsilateral vs. control. **p < 0.05 contralateral vs. i*p*silateral and control. Instrumental conditioning experiments were conducted twice, with concordant results. A version of **g** with individual mice represented is provided in Suppl. Fig. 6c.

To begin to identify projections that might be important for MC4R-controlled behavior, we returned to our original population of experimentally bred response-inflexible mice and quantified dendritic spine densities on distal dendritic segments – considered highly labile^29^ – as a general measure of neural plasticity, akin to measuring immediate-early gene expression. Densities on excitatory layer V OFC neurons (ventrolateral subregion) were higher in response-inflexible mice *vs*. age-matched controls (Fig. 4a), but not in prelimbic, infralimbic, or hippocampal CA1 regions (Fig. 4a).

Next, we classified dendritic spines into their primary subtypes, including mushroom-shaped spines, which are considered mature, stable, and synapse-containing, compared to thin- or stubby-shaped spines, which by contrast are immature and functionally variable^30^. Mice that failed to inhibit responding when pellets were delivered noncontingently (contingent/noncontingent scores ≤1) had more immature, thin-type spines. Meanwhile, mice that *did* inhibit responding (scores > 1) were considered resilient (Fig. 4b) and had more mature, mushroom-shaped spines on OFC neurons (Fig. 4c). Thin-type spine densities also correlated with response strategies in 2 independent cohorts of mice (Fig. 4d). Thus, poor response inhibition is associated with immature spine types, while successful strategy shifting is associated with mature spine types in the OFC, leading to the hypothesis that the OFC is part of a network controlling response inhibition.

OFC-to-DMS inputs are organized largely ipsilaterally in the brain, including in mice^31^. We took advantage of these segregated projections to use a “disconnection” design to test the possibility that connections with the OFC were necessary for the behavioral flexibility conferred by silencing *Mc4r* in the DMS. Here, we reduced *Mc4r* unilaterally in one DMS and placed Gi-coupled Designer Receptors Exclusively Activated by Designer Drugs (DREADDs) unilaterally in one OFC (Fig. 4e). When infusions are ipsilateral and the DREADDs ligand Clozapine N-oxide (CNO) is delivered, one DMS lacks *Mc4r*, which *should* improve response inhibition, but it is devoid of the typical OFC signal. We thus anticipated that this group would resemble mice with control viral vectors. Meanwhile, in the contralateral (“asymmetric”) group, mice also experience unilateral OFC inactivation, but the healthy OFC is projecting to an *Mc4r* knockdown DMS. If these OFC-to-DMS connections can account for response inhibition following *Mc4r* knockdown, we reasoned that this group should be *better* able to inhibit responding when food is delivered noncontingently, relative to the control groups.

OFC-targeted infusions were largely contained within the ventrolateral region, and terminals were detected in the DMS, overlapping with areas in which *Mc4r* was reduced (Fig. 4f). Viral vector spread in the knockdown group was comparable to the prior figure and contained within the DMS (Suppl. Fig. 4). In the control group, some spread into the ventral striatum was noted (Fig. 4f), but did not have obvious consequences. Groups did not differ during response training, conducted in the absence of CNO (Fig. 4g).

When one familiar behavior failed to be reinforced, and instead, pellets were delivered noncontingently, the contralateral group generated the lowest response rates (Fig. 4g), differing from mice bearing control viral vectors in the final three time bins. Importantly, while the ipsilateral mice responded less than control mice during the third time bin, this difference was transient and they ultimately were not as adept at inhibiting nonreinforced behaviors as the contralateral group (Fig. 4g). These patterns together suggest that response inhibition conferred by *Mc4r* silencing in the DMS requires input from the ventrolateral OFC.

## Discussion

Here we trained mice to generate two responses in operant conditioning chambers for food reinforcers. We then uncoupled the predictive relationship between one response and its outcome by providing food pellets noncontingently, and responding was not reinforced. Typically, mice inhibit that response and favor the other, but individual differences exist, such that a minority of mice here failed to readily inhibit familiar behaviors, even when those behaviors were not explicitly reinforced. We bred these mice, generating offspring with the same tendencies. By thereby generating large numbers of mice that failed to readily inhibit reward-seeking behaviors, we were able to resolve correlations between MC4R in the DMS and response strategies. These patterns led to experiments revealing that MC4R presence in the DMS propels reward-seeking behavior, while reducing MC4R expedites response inhibition, an effect that relies, at least in part, on OFC input.

What might account for transgenerational response biases? We used transgenic mice expressing YFP and bred on an inbred C57BL/6 background, which makes genetic variation unlikely. Experimental mice were compared to the offspring of other C57BL/6 mice bred in our lab that had also been behaviorally tested; thus, epigenetic effects of behavioral testing, writ large, are also unlikely. Conceivably, other epigenetic effects and/or familial factors could play a role. We did not observe gross differences in maternal behavior when quantified during the light cycle (Suppl. Fig. 5), but potentially, maternal care differed between groups during the dark cycle, which could propel behavioral differences in adulthood. These and other possibilities could be investigated in the future. Our present goal was to amplify individual differences in response inhibition capacity by breeding response-inflexible mice and thereby creating a tool by which to better understand the neurobiology of instrumental behavior.

Several independent investigations indicate that the DMS is necessary for rodents to modify familiar reward-seeking behaviors^1–5^. These observations motivated us to measure synaptic markers in the DMS of experimentally bred, response-inflexible mice. PSD-95, a post-synaptic marker associated with synaptic strength^32^, was lower than in typical mice. Meanwhile, synaptophysin, a presynaptic marker associated with synapse density^33^, was unaffected. Less PSD-95 thus likely reflects weaker excitatory synapses in the DMS, rather than the loss of inputs from extra-striatal regions, per se.

Striatal CNPase, a marker of mature oligodendrocytes, was also quantified. Once considered merely an insulator of neurons, oligodendrocytes are dynamic, sensitive to stressors, alcohol, motor skill learning, and electrical and synaptic activity^34–37^. It appeared that experimental breeding reduced CNPase, but this effect did not survive correction for multiple comparisons.

Next, we quantified MC4R, the high-affinity receptor for α-MSH, a peptide produced by proopiomelanocortin (POMC)-expressing neurons in the arcuate nucleus of the hypothalamus. Levels of MC4R in the DMS correlated with response strategies, such that high levels were associated with pursuit of familiar response strategies. Meanwhile, mice with low levels demonstrated response flexibility, reminiscent of evidence that low *Mc4r* confers resilience to compulsive-like behavior^17^.

These patterns led us to test MC4R function in the DMS using viral-mediated site-selective *Mc4r* gene silencing. Reducing MC4R expediated response inhibition, enriching the capacity of mice to restrain behaviors that were not reinforced. We also tested the capacity of mice to modify behavior based on reward value. We reasoned that if silencing *Mc4r* enriches response plasticity, then *Mc4r*-deficient mice would more rapidly inhibit responding when a reward lost value. Indeed, inhibiting MC4R in the DMS conferred response flexibility, since *Mc4r*-deficient mice more rapidly inhibited responding when foods were devalued than control mice.

Why might melanocortin-MC4R action in the DMS propel familiar reward-seeking behaviors? In the striatum, MC4R preferentially expresses on dopamine D1 receptor (D1R)-containing medium spiny neurons (MSNs)^15,25,38^. MC4Rs, like D1Rs, are positively coupled to the cAMP second messenger cascade^18,39^, and thus can enhance D1R function^40^. D1R stimulation is necessary for learning new skills^6^, and D1R+MSNs in the DMS is involved in the development of goal-directed action strategies^10^ – a process that requires inhibiting unproductive behaviors – and recalling memories linking actions and outcomes^41^. *Mc4r*-null mice are delayed in learning to nose poke for food, and restoration of MC4R in D1R-containing cells reinstates this capacity^25^. Possibly, MC4R + D1R stimulation synergistically attunes mice to actions predictive of reward, particularly when learning new tasks, thus propelling those actions. Conceivably, high levels of MC4R (as in inflexible mice) could overly drive reward-seeking behaviors at the expense of adaptive response plasticity.

Why might reducing Mc4r facilitate response inhibition? MC4Rs regulate GluA2 AMPAR subunit availability at the membrane. α-MSH-MC4R binding triggers GluA2 internalization^28^. Meanwhile, decreasing MC4R enhances glutamatergic signaling in the striatum^17^. Given that dopamine agonists increase POMC, the precursor for α-MSH^42^, and cocaine increases striatal α-MSH content^43^, rewarding events may result in α-MSH-MC4R binding. This binding would cause GluA2-AMPAR internalization, decreasing the synaptic sensitivity of DMS MSNs to cortico-striatal glutamatergic afferents, which otherwise trigger response plasticity and suppression in many contexts^44–46^. Thus, reducing MC4R levels or activity would increase sensitivity to cortico-striatal projections that might trigger response inhibition when adaptive.

Implicit in this model is that the apparent “pro-flexibility” effects of *Mc4r* silencing depend on glutamatergic input to the DMS. We attempted to identify likely sources of inputs, first returning to our original experimentally bred response-inflexible mice. We quantified dendritic spines on terminal dendrites in multiple brain regions, because terminal dendrites are highly plastic and can be viewed as a general proxy of neural plasticity – conceptually similar to measuring immediate-early gene expression^29^. Response-inflexible mice had higher densities of thin-type dendritic spines on excitatory neurons in the OFC, which are unstable and typically pruned with instrumental conditioning^47^. Meanwhile, dendrites from response-flexible mice hosted more mature, mushroom-shaped spines. Notably, we found no obvious group differences on dendrites in the PL, IL, or hippocampal CA1, even while neuronal structural plasticity in the PL, for example, has been associated with instrumental response strategies in the same task^48^. Further, stress-induced failures in response flexibility in a very similar task are associated with dendritic spine loss on proximal branches of apical PL dendrites (and also loss of terminal branches^49^). A key difference, though, is that the majority of investigations into dendritic spine densities, particularly ex vivo investigations, focus on dendritic segments at some fixed distance from the soma, while we instead imaged distal, terminal tufts, which are considered more plastic and subject to in-the-moment events and stimuli. Putting the pieces together, then, we might imagine that previously reported modifications in the PL could reflect long-term changes (for instance, associated with initially learning action-reward contingencies), rather than acute effects (for instance, of detecting the violation of learned rules).

We next hypothesized that excitatory plasticity in the OFC may be involved in response flexibility conferred by moderating MC4R tone in the DMS. The OFC and DMS are connected by unidirectional projections organized largely ipsilaterally in the brain^31^. We capitalized on this anatomical organization and infused into the OFC of one hemisphere inhibitory Gi-coupled DREADDs. In the ipsilateral or contralateral DMS, *Mc4r* was reduced. In the ipsilateral condition, one DMS had less *Mc4r*, but was deprived of typical OFC input – we anticipated that these mice would resemble control mice (those bearing control viral vectors). Meanwhile, in the contralateral condition, mice had the same manipulations, but the DMS that had less *Mc4r* received input from the OFC. If OFC input on striatal neurons with low MC4R optimizes adaptive response inhibition – as we predicted – we expected that this group would be best able to inhibit responding. This was indeed the case. Thus, reducing *Mc4r* appears to facilitate response plasticity at least in part via OFC input.

A final note is that MC4R levels in the ventral striatum did not correlate with response patterns here. This outcome was interesting, given that the ventral striatum is more strongly innervated by α-MSH-containing projections from the arcuate nucleus than the DMS^28^. MC4R antagonism and gene silencing in the ventral striatum mitigate cocaine-seeking, anhedonic-like, and compulsive-like behaviors^15–17,28^, and ventral striatal MC4R controls approach and avoidance of both appetitive and aversive stimuli^50^. Altogether, then, it appears that ventral striatal MC4R stimulation promotes drug seeking and compulsion, while MC4R activity in the DMS appears to propel reward-seeking behaviors. Meanwhile, inhibiting MC4R appears to combat drug seeking and anhedonic-like behavior and promote the capacity for behavioral inhibition – qualities that could be favorable in treating addictions and other illnesses.

## Methods

### Subjects

Initial experiments bred mice with particular behavioral traits and tested their offspring. These mice were maintained on a C57BL/6 background and expressed *Thy1*-driven YFP-H^51^ (Jackson Labs), allowing us to visualize neurons and enumerate dendritic spines in some experiments. In experiments in which we manipulated *Mc4r*, mice were homozygous for a ‘floxed’ *Mc4r* gene^24^ (Jackson Labs). These mice were maintained on a mixed C57BL/6J-129S1/SvImJ background.

Mice were weaned from the dam at or soon after postnatal day (P) 21 and housed in single-sex cages with siblings or unrelated mice of the same age. Mice were maintained on a 12-h light cycle (0700 on) and provided food and water ad libitum except during food-reinforced behavioral testing when food was restricted to motivate responding. Experiments used both sexes. Sex differences were observed in one experiment, and sex was accordingly included as a factor in statistical analyses. Procedures were approved by the Emory University IACUC.

### Ages of mice at testing

Behavioral testing used to identify mice for breeding was initiated between postnatal days (P) 27-30. Once identified, mice were paired with opposite-sex counterparts at or soon after P56. In other experiments, animals were ≥P56 at the time of testing and behaviorally naïve.

### Test of action strategies

Mice were food restricted to motivate food-reinforced responding. In young mice, body weights were maintained at 100% of the expected growth curves for C57BL/6 mice (Jackson Labs) to maintain animals’ health. In mature (≥P56) mice, body weights dropped to ~93% of their free-feeding weight. Operant conditioning chambers (Med-Associates) were equipped with 3 nose poke ports, as well as a separate food magazine. Responding on 2 of the ports was reinforced with food pellets (20 mg, Bio-serv) using a fixed ratio 1 (FR1) schedule of reinforcement. Up to 30 pellets were available for responding on each port, resulting in 60 pellets/session. Sessions ended when 60 pellets were delivered or at 70 min, whichever came first. Mice did not develop side or pellet preferences, and response acquisition curves represent both nose poke responses/min. Nose poke training occurred over 7–9 days, with 1 session/day.

Next, one port was occluded, and responding on the other had no programmed consequences. Instead, pellets were delivered into the magazine at a rate matched to each animal’s reinforcement rate from the previous day (i.e., pellets were delivered “for free”). Thus, the response-reward relationship linking this nose poke and reward was violated, which typically causes mice to cease responding at this port. This session is referred to as the “noncontingent” session. A 25-min “contingent” session served as a control; here, the other nose poke port was available, and responding remained reinforced according to an FR1 schedule of reinforcement. The location of the “noncontingent” port within the chamber was counter-balanced.

In mice screened for breeding, we also assessed responding the next day, during a brief probe test, in which both ports were available for 10-min. Responses were recorded but not reinforced. Groups did not differ during this phase, so responding by two cohorts is shown, but not for others.

### Breeding strategy

Mice were paired for breeding if they fulfilled 2/3 of the following criteria: (1) >20% of total responses occurred on the inactive port during response training; (2) they failed to inhibit responding during the “noncontingent” session relative to “contingent” session; or (3) they failed to prefer the “contingent” nose poke during the probe test. We first behaviorally characterized 52 mice. Fifteen mice created the parental generation, and their offspring created the F1 generation, which was then tested as its parents were. They were compared to same-age, same-strain mice whose parents had also been behaviorally tested. Mice were again selected for breeding based on the above-described criteria, and their offspring created the F2 generation, which was tested as its parents were. Following the F1 generation, care was taken to ensure that siblings were not bred. These mice are represented in Fig. 1. For subsequent studies, experimental mice were the offspring of mice that had been selected for breeding as described above. Control mice were age- and strain-matched mice bred in our colony.

### Reinforcer devaluation

One group of mice tested in the above-described behavioral assay was next used in a devaluation experiment. Mice had one re-training session according to an FR1 schedule of reinforcement for 70 min to reinstate responding on both nose poke ports. As above, responding on two ports was reinforced with either a grain-based or chocolate-flavored pellet (20 mg, Bio-serv). Mice did not display systematic pellet preferences, as can be seen in the associated figure.

CTA was then used to decrease the value of one of the pellets. Mice were placed individually in clean cages with free access to one of the two pellets. After 60 min, mice were injected with lithium chloride (LiCl; 0.15 M in saline, 4 ml/100 g, i.p., Sigma), which induces temporary gastric malaise. The following day, mice were given ad libitum access to the other pellet for 60 min, followed by a vehicle injection (NaCl). Mice experienced 6 pairing sessions/pellet across 12 days. Pellet intake was measured and compared between groups and conditions.

Our hypothesis was that DMS-selective *Mc4r* knockdown would enhance the ability of mice to inhibit responding. To test this possibility, we placed mice in the conditioning chambers for a probe test after only 3 CTA pairings (15 min, conducted in extinction), before mice developed robust CTA. The idea was that this timing would allow us the resolution to detect enhanced performance, if it indeed existed. The probe test was then repeated following all 6 pairings to confirm that CTA would, with sufficient training, reduce responding for the LiCl-paired pellet as expected.

After both probe tests, mice were placed individually in a clean cage with an abundant, equivalent supply of both pellets, allowing them to freely consume pellets. Remaining pellets were measured after 60 min to quantify ad libitum intake. The point of this measure is to confirm that CTA is effective, and thus, behavioral responding in the probe test reflects the propensity (or not) of mice to modify behaviors based on goal features.

### Delay discounting

This procedure was adapted from Adriani and Laviola^52^. Operant conditioning chambers (Med-Associates) were equipped with 2 nose poke ports, as well as a separate food magazine. For 9 30-min sessions, instrumental training occurred according to an FR1 schedule of reinforcement. Responding in 1 port resulted in the delivery of 1 pellet (20 mg grain-based pellets; Bio-Serv). Responding in another port resulted in the delivery of 5 pellets, paired with a 1-s flash of the house light. Responding in either port was followed by a 25-s time-out, during which responses were recorded but not reinforced. For the extent of the time-out period, a separate light was illuminated. Mice were considered to have acquired the responses when they displayed a preference (>50% responses) for the larger reinforcer over 2 consecutive days.

After training, the delay phase commenced, such that responding for the large reinforcer triggered a delay before reinforcer delivery. The delay length remained constant within sessions and increased between the daily sessions. Delay lengths were 10 s, 20 s, 30 s, 45 s, 60 s, 80 s, and 100 s. The house light was illuminated during the delay. Responding for the large vs. small reinforcers were compared, as were responses during the time-out periods.

### Intracranial surgery and viral vectors

*Mc4r*-flox mice were anesthetized via ketamine (100 mg/kg, i.p.) and dexmedetomidine (0.5 mg/kg, i.p.). Mice were administered the analgesic meloxicam (5 mg/kg, s.c.) and revived using atipamezole (1 mg/kg, i.p.). Drugs were dissolved in saline and administered in a volume of 1 ml/100 g.

For DMS infusions, adeno-associated viral vectors (AAV8) expressing Green Fluorescence Protein (GFP) ± Cre-Recombinase (Cre) with a CamKIIα promotor were supplied by the UNC Viral Vector Core. Viral vectors were infused at a rate of 0.1 µl/min, with a total volume of 0.5 µl, at +0.5 mm anteroposterior (AP), −4.5 mm dorsoventral (DV), and ±1.6 mm mediolateral (ML) relative to Bregma. The micro-syringe was left in place for 5 min following infusion.

In some experiments, viral vectors were also delivered to the OFC. For OFC infusions, mice received unilateral infusions of AAV5-CaMKIIα-mCherry ± hM_4_D(Gi) (UNC Viral Vector Core) in the ventrolateral OFC (0.5 µl/infusion over 5 min at AP + 2.6, ML ± 1.2, DV-2.8). Simultaneously, they received unilateral infusions of AAV ± Cre into the DMS as above. Infusions were either ipsilateral or contralateral. The micro-syringes were left in place for 5 additional min prior to withdrawal and suture. The ipsilateral and contralateral control groups (i.e., mice that received the control viral vector in the OFC and DMS) did not differ and were combined for statistical and graphical purposes. For general description of DREADDs, see Urban and Roth^53^. Mice were allowed ≥3 weeks for recovery and viral vector expression.

### CNO administration and timing in DREADDs experiments

Mice with DREADDs were trained to nose poke as described, and they received injections of saline 30 min before the instrumental training sessions to habituate them to injection stress. Then, CNO (Sigma) was delivered at 1 mg/kg, i.p., dissolved in 2% DMSO and saline (1 ml/100 g) 30 min before the “noncontingent” session of our procedure. All mice received CNO, regardless of condition, to equally expose animals to any unintended consequences of CNO^54^.

### Assessments of food intake

To determine whether reducing *Mc4r* in the DMS impacted free-feeding behaviors, we reduced *Mc4r* in the DMS bilaterally, and we then assessed food intake used established methods^55–57^: Mice were singly housed for 2 weeks prior to the experiment. Mice were given ad libitum standard chow and water. Baseline body weight was collected, and then body weight and food intake were subsequently measured daily for 7 days, 3 h after lights on.

### Assessments of maternal care

We adapted a procedure reported by Heath et al.^58^. Pregnant dams were monitored daily and the day of birth was designated P0. Then, maternal behavior was observed 7 times over the 3-week post-partum period. Observations occurred 2–3 h before lights off for 10 min/session. Maternal behavior was recorded every 30 s. Dams were recorded as being engaged in: licking and grooming of pups, nest arranging or snout contact with nesting pups, passive nursing, arched-back nursing, and no contact with pups. Arched-back nursing was scored when mice engaged in effortful crouching over the pups, which were gathered beneath her. Other nursing behavior was scored as “passive nursing.” Care was taken to avoid observing mice on days when cages had been changed. Control dams were same-strain dams that had given birth within 48 h of the experimental dam. In one case, 2 experimental dams gave birth at the same time and were matched with a single control dam. The results of these experiments are reported in Suppl. Fig. 5.

### Histology

Following testing, mice with viral vectors were euthanized either by decapitation following brief anesthesia with isoflurane or more commonly, by deep anesthesia with ketamine/xylazine (100 and 10 mg/kg, i.p.), followed by intracardiac perfusion with chilled saline and 4% paraformaldehyde. Brains were soaked in 4% paraformaldehyde for 48 h, then transferred to 30% w/v sucrose, and sectioned into 40–50-µm-thick sections on a freezing microtome. Tissues were plated, then imaged using a fluorescence microscope. If infusions were not contained within the DMS or OFC, mice were excluded.

### Immunoblotting

Mice had been trained and tested in the first behavioral task described above. They were returned to free-feeding and left undisturbed for roughly 1 week. Then, they were briefly anaesthetized with isoflurane and euthanized by rapid decapitation, and brains were extracted and frozen at −80 °C. Brains were sectioned into 1 mm coronal sections using a chilled brain matrix, and punches aimed at the DMS and ventral striatum were extracted using tissue corers. Ventral striatal tissue extractions took care to avoid the anterior commissure, and some were unintentionally lost. Tissues were homogenized by sonication in lysis buffer [200 µl: 137 mM NaCl, 20 mM tris-Hcl (pH = 8), 1% NP-40, 10% glycerol, 1:100 Phosphatase Inhibitor Cocktails 2 and 3, 1:1000 Protease Inhibitor Cocktail (Sigma)], and stored at −80 °C. Protein concentrations were determined using a Bradford colorimetric assay (Pierce).

Equal amounts of protein (15 μg) were separated by SDS-PAGE on 7.5% or 4–20% gradient Tris-glycine gels (Bio-rad). Following PVDF membrane transfer, blots were blocked with 5% nonfat milk or 5% BSA for 1 h. Membranes were incubated with primary antibodies at 4 °C overnight and then incubated in horseradish peroxidase secondary antibodies for 1 h. Primary antibodies were PSD-95 (Ms, Cell Signaling #3450, 1:1000), Synaptophysin (Rb, Abcam #32127, 1:20,000), CNPase [Ms, Millipore (multiple tested), 1:1000], MC4R (Rb, Abcam #150419, 1:1000), Tau (Rb, Cell Signaling #46687; 1:1000), Tyrosine hydroxylase (Rb, Sigma #AB152; 1:1000), GluN2B (Ms, Novus Biologicals #NB100-74475; 1:500), Alpha-tubulin (Rb, Cell Signaling #3873; 1:1000), and Calmodulin (Rb, Cell Signaling #35944; 1:1000).

Immunoreactivity was assessed using a chemiluminescence substrate (Pierce) and measured using a ChemiDoc MP Imaging System (Bio-rad). Densitometry values were individually normalized to the corresponding loading control (HSP-70; Ms, Santa Cruz Biotechnology #7298, 1:5000), which did not change as a function of breeding, and then normalized to the control sample mean from the same membrane in order to control for fluorescence variance between gels.

### Dendritic spine imaging and reconstruction

Mice had been trained and tested in the first behavioral task described above. Roughly 24 h later, mice were briefly anaesthetized by isoflurane and euthanized by rapid decapitation. Brains were submerged in chilled 4% paraformaldehyde for 48 h, then transferred to 30% w/v sucrose, and sectioned into 40–50-µm-thick sections on a freezing microtome. Mice carried *Thy1*-driven YFP, resulting in YFP expression in layer V cortical neurons and hippocampal CA1. Z-stacks were collected with a 100 × 1.4 numerical port objective using a 0.1 µm step size on a spinning disk confocal (VisiTech International) on a Leica microscope. 6–10 segments/mouse were imaged. They ranged from 19 to 31 µm in length. Experimenters were blind to group in all experiments.

#### Experiment 1. Multi-site quantification of dendritic spine densities

Dendritic segments in the prelimbic prefrontal cortex (PL), infralimbic prefrontal cortex (IL), ventrolateral OFC, and dorsal hippocampal CA1 were imaged, with The Mouse Brain in Stereotaxic Coordinates^59^ as reference. We endeavored to image terminal segments, which are considered highly plastic. Dendritic spines were manually counted, normalized to the length of the dendrite (spines/µm), and each mouse contributed a single value (its mean density) to comparisons.

#### Experiment 2. Defining individual differences in dendritic spine densities and morphologies

Response inhibition in our decision-making task triggers the elimination of thin-type dendritic spines in the ventrolateral OFC, increasing the proportion of mushroom-shaped spines^47^. We thus characterized dendritic spine morphologies in several mice that had been behaviorally characterized. We separated mice by those that failed to inhibit responding when pellets were delivered noncontingently (contingent/noncontingent scores ≤1) vs. mice that did inhibit responding (scores > 1) for comparisons. Using ImageJ, dendritic spines were enumerated. Also, dendritic spine heads were traced at the widest point, and the length of each spine was collected, allowing us to classify spines into their primary subtypes. Dendritic spines with heads ≥0.35 µm in diameter and > 0.45 µm in length were considered mushroom-like, while dendritic spines that were > 0.45 µm in length with heads smaller than 0.35 µm in diameter were considered thin-type. Spines < 0.45 µm in length were considered stubby. Again, each mouse, rather than each dendrite, was considered an independent sample.

### Statistics and reproducibility

Our initial experiment contained 52 mice, each considered an independent sample. The parental generation from this experiment created the F1 generation. A male and female from each F1 litter were tested. In the rare instances that the litters contained only 1 sex, then 2 mice of the same sex were tested. Here, each litter was considered an independent sample, reflecting the mean of the 2 mice tested from that litter. The same approach was taken with the F2 generation. With the F3 generation, we tested all mice in a litter, then calculated the proportion of mice that were able to inhibit a nonreinforced response (that is, they generated at least one fewer response when pellets were delivered noncontingently relative to a session of the same duration when pellets were delivered contingently). Each litter contributed one proportion value to the comparison. In subsequent experiments, experimentally bred mice were derived from independent litters and treated as independent samples.

In our initial experiment (Fig. 1), response rates during training were compared by ANOVA with repeated measures, then response rates between the contingent vs. noncontingent response conditions were compared by paired *t*-tests. Proportions in Fig. 1g were compared by unpaired t-test. In subsequent experiments, response rates, body weights, food intake, and maternal care counts were compared by ANOVA, with repeated measures when appropriate. In the case of interactions or main effects between >2 groups, post-hoc comparisons used Tukey’s or Student’s tests; all possible comparisons were made, and any significant differences are reported. Comparisons were two-tailed unless otherwise noted. Alpha was set at 0.05.

Western blot values were compared by or 1- or 2-factor ANOVA. Dendritic spine densities were compared by unpaired *t*-tests or 2-factor ANOVA. These exploratory comparisons (Figs. 2, 4) were subject to the Benjamini–Hochberg Procedure for correcting for multiple comparisons, with a false discovery rate of 5%. Posthoc *t*-tests in Fig. 4c were one-tailed due to a priori hypotheses based on previously reported dendritic spine pattens in typical mice performing the same task^47^.

Western blot values and dendritic spine densities were also compared by linear regression against response preference scores – the response rates in the contingent/noncontingent conditions. Scores >1 reflect inhibition of the nonreinforced behavior, while scores at ≤1 reflect no change in response strategies relative to training. Western blots were subject to replication, with concordant results. Each mouse, rather than each technical replicate, was considered an independent sample.

Exclusions: Values >2 standard deviations outside of the mean were considered outliers. One mouse from each group in the “disconnection” experiment in the final figure generated multiple outlying values during training and was excluded. Proportion data were not subject to outlier analysis. Any mice with misplaced viral vectors were also excluded. Finally, 1 mouse in the delay discounting procedure did not nose poke and was excluded. Final *n*’s are reported in the figure captions. SPSS v.28 and SigmaPlot v.11 and 14.5 were used to analyze data.

### Reporting summary

Further information on research design is available in the Nature Research Reporting Summary linked to this article.

## Supplementary information

Supplementary Information

Description of Additional Supplementary Files

Supplementary Data 1

Reporting Summary

## Data Availability

Data and uncropped gels are provided in the Supplementary Data 1 file and Suppl. Fig. 7, respectively.
