# Supplementary material for: Inter-individual variability amplified through breeding reveals control of reward-related action strategies by Melanocortin-4 Receptor in the dorsomedial striatum
Source: Commun Biol. 2022 Feb 8;5:116. doi: 10.1038/s42003-022-03043-2 (PMC8825839; doi:10.1038/s42003-022-03043-2)
Supplement: Supplementary file 1 — Supplementary Information [file 42003_2022_3043_MOESM1_ESM.pdf]

Suppl. fig.1. Memory for the noncontingent condition is intact in the offspring of experimentally-bred mice.

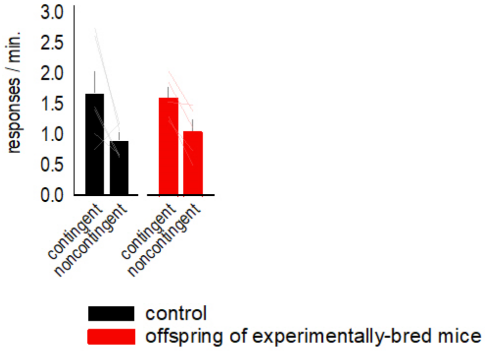

Consistently, the offspring of experimentally-bred mice demonstrated intact preference for the response associated with contingent pellet delivery when tested the day following the contingency violation. This pattern indicates that memory for the contingent *vs.* noncontingent conditions was intact, despite poor response inhibition the previous day. The F2 generation from figure 1 of the main text is shown as an example [control t_5=2.48, p=0.056, n=6; offspring t_4=4.1, p=0.015, n=5]. Each litter was considered an independent sample for statistical comparisons by paired t-tests between conditions. Bars represent means + SEMs, and individual lines represent individual litters.

Suppl. fig.2. Delay discounting was unaffected by experimental breeding.

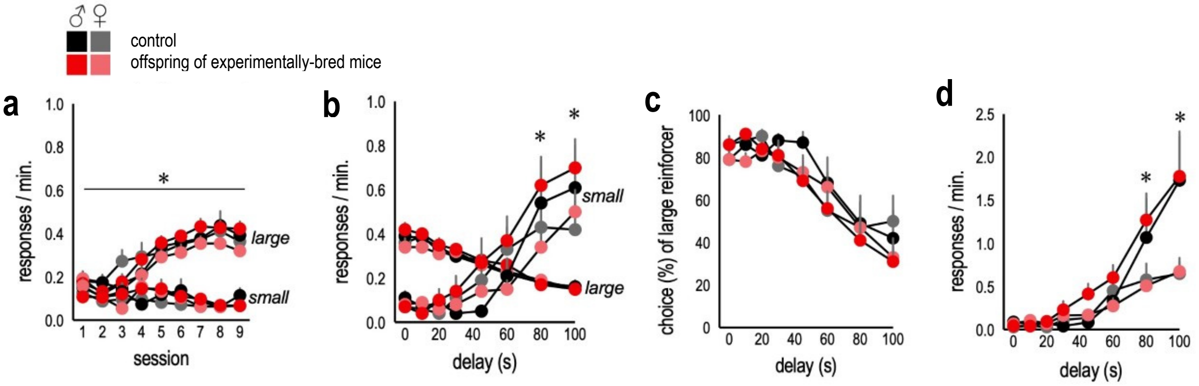

(a) Mice of both sexes respond more for large *vs*. small reinforcers over time [main effect of reinforcer size $F_{(1,43)}$=178.1, p<0.001; main effect of session $F_{(8,344)}$=154.2, p<0.001; reinforcer size x session interaction $F_{(8,344)}$=256.4, p<0.001; no main effects or interactions with reinforcer size or sex or group Fs<1; no reinforcer size x sex x group interaction $F_{(1,43)}$=2.5, p=0.12; no session x group interaction $F_{(8,344)}$=1.2, p=0.30; no session x sex interaction $F_{(8,344)}$=1.4, p=0.19; no session x group x sex interaction $F_{(8,344)}$=1.5, p=0.17; no reinforcer size x session x group or reinforcer size x session x group x sex interaction Fs<1; no reinforcer size x session x sex interaction $F_{(8,344)}$=1.4, p=0.21].

(b) When delays are introduced between responses and large reinforcers, mice shift preference. We detected no group differences [main effect of delay $F_{(7,294)}$=13.5, p<0.001; delay x reinforcer size interaction $F_{(7,294)}$=43.0, p<0.001; no main effect of reinforcer size $F_{(1,42)}$=2.7, p=0.11; no main effect of sex $F_{(1,42)}$=2.11, p=0.15; no delay x sex interaction $F_{(7,294)}$=1.40, p=0.21; no delay x group x sex interaction $F_{(7,294)}$=1.22, p=0.29; no reinforcer size x delay x sex interaction $F_{(7,294)}$=1.54, p=0.16; no reinforcer size x delay x group x sex interaction $F_{(7,294)}$=1.10, p=0.36; no main effect of group or other interactions of group, sex, reinforcer size, or delay, Fs<1]. *p<0.05 preference for the small reinforcer.

(c) The same data from B are converted to preference for the large reinforcer [main effect of delay $F_{(7,294)}$=37.9, p<0.001; no effect of group or sex, or interaction of group or sex with delay Fs<1; no pellet x sex x group interaction $F_{(7,294)}$=1.5, p=0.16].

(d) As the delay increased, non-reinforced responding increased for all groups [main effect of delay $F_{(7,294)}$=25.0, p<0.001]. Males engaged in more non-reinforced responding than females

with increasing delay, reaching levels of non-reinforced responding over twice that of females [main effect of sex $F_{(1,42)}=4.9$, $p=0.03$; sex x delay interaction $F_{(7,294)}=5.5$ $p<0.001$; no effect or interactions with group, *F*s<1]. n=10 control male, 16 male offspring of experimentally bred mice, 11 control female, 9 female offspring of experimentally bred mice. Symbols represent means + SEMs. *$p<0.05$.

Suppl. fig.3. No alterations in several proteins due to experimental breeding, including in behaviorally-naïve mice.

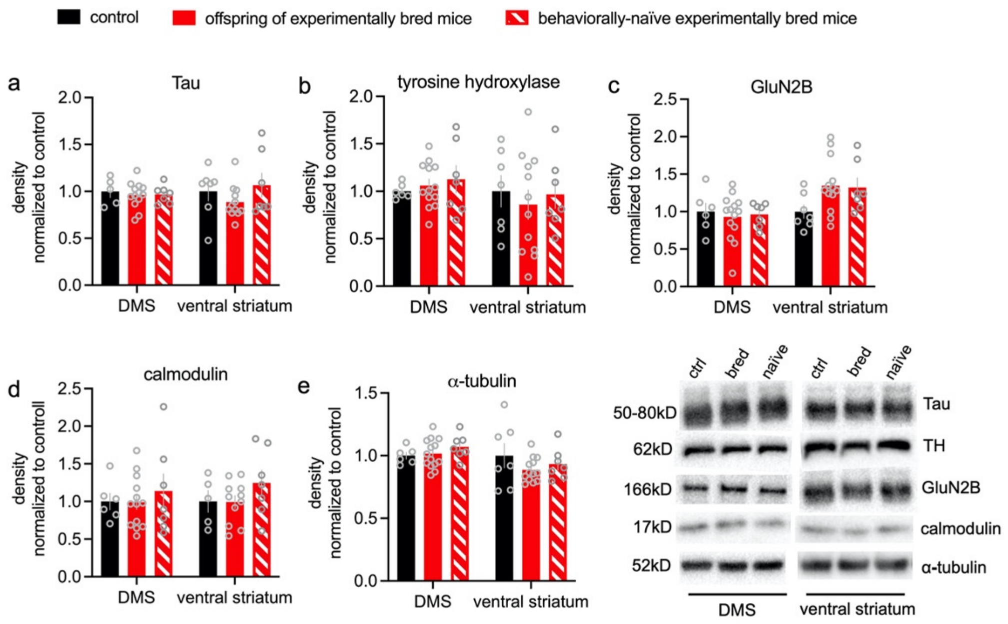

In addition to those in the main text, we quantified several other proteins in the striatum of offspring of experimentally-bred mice, including both naïve and behaviorally-tested mice, and both the DMS and ventral striatum. Control mice were behaviorally-tested, same-age, same-strain mice bred in our lab. These proteins were (a) Tau (both F<1), (b) tyrosine hydroxylase (TH; both F<1), (c) GluN2B (DMS F<1); ventral striatum [$F_{(2,24)}$=2.8, p=0.08]), (d) calmodulin (both F<1), and (e) α-tubulin (DMS [F<1]; ventral striatum [$F_{(2,24)}$=1.18, p=0.33]). Bars represent means + SEMs, and symbols represent individual mice. Representative blots are adjacent. All proteins were detected at expected molecular weights, and gels were run at least twice, with concordant results. Control n=5-7, offspring of experimentally-bred mice n=12-14, naïve offspring n=7.

Suppl. fig.4. Cre-expressing viral vector infusion sites corresponding to main text figure 4.

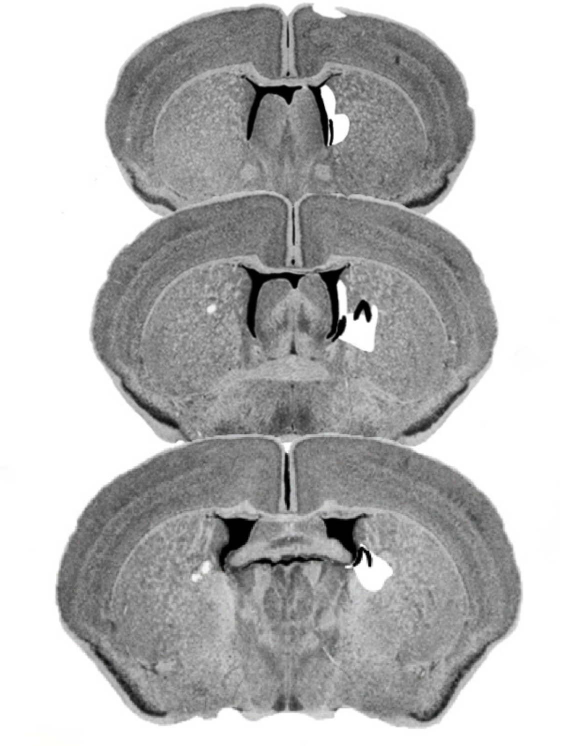

Cre-expressing viral vectors corresponding to main text figure 4 are summarized. White represents the largest (and typical) viral vector spread, and black the smallest. (Most mice are consistent with the white traces.) Images from the Mouse Brain Library[1] were used to create these images.

Suppl. fig.5. Experimental breeding did not obviously affect maternal behaviors.

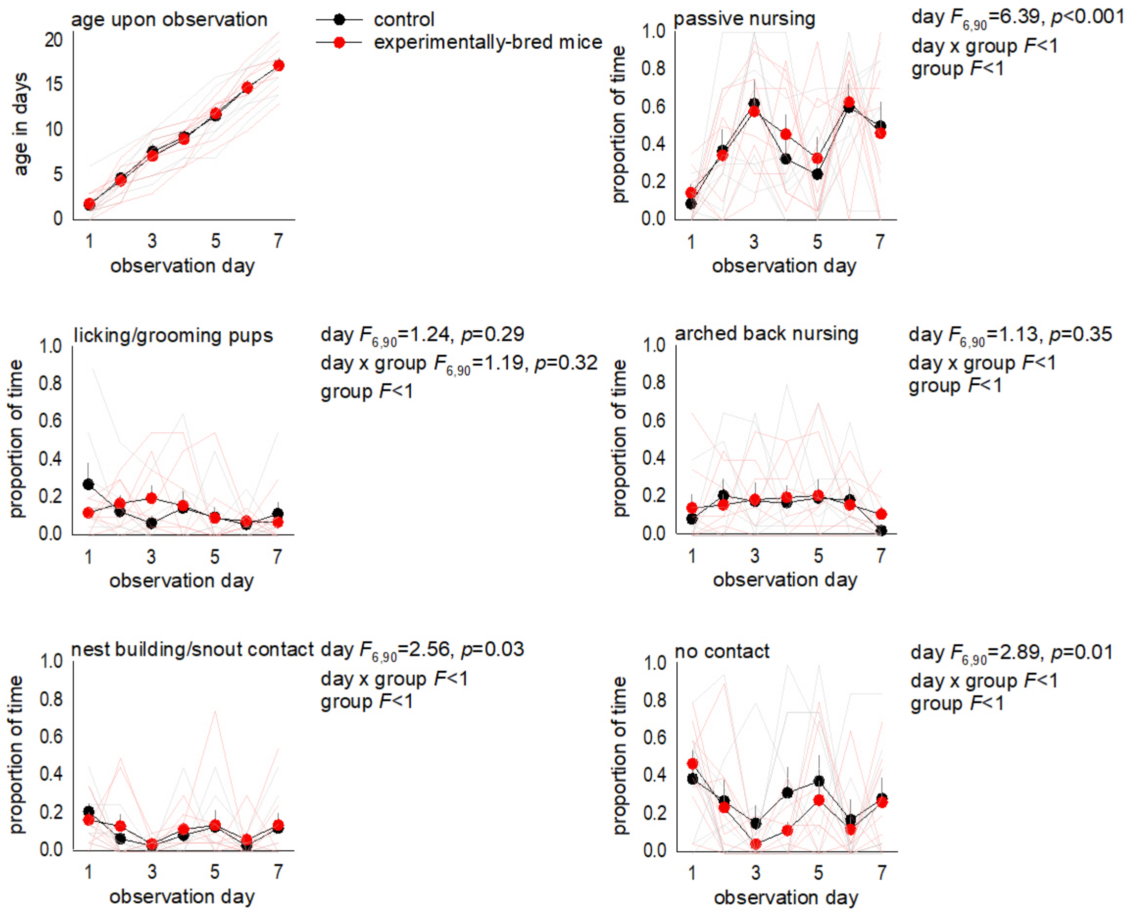

We observed dams across the 3-week post-partum period. The average age of the pups upon observation is represented at top left. We found no group differences in the proportion of time spent engaged in: arched-back nursing; passive nursing; licking and grooming; nesting and snout contact in the nest; and no contact. Statistics are provided at right. Time spent nesting and in snout contact generally declined, consistent with prior reports regarding the same strain of mice[2]. Passive nursing generally increased with time, in agreement with evidence that nursing not occurring in the arched-back position increases with the age of the pups[3]. Time outside of the nest varied non-systemically. This pattern might be attributable to the timing of our observations – occurring as the dark cycle approached, a time when activity in and out of the nest fluctuates in C57BL/6 mice[2]. Symbols represent means + SEMs. n=8 control dams and 9 experimentally-bred dams. Symbols represent means + SEMs, and light lines represent individual mice.

Suppl. fig.6. Figures corresponding to main text, with individual mice shown in light lines.

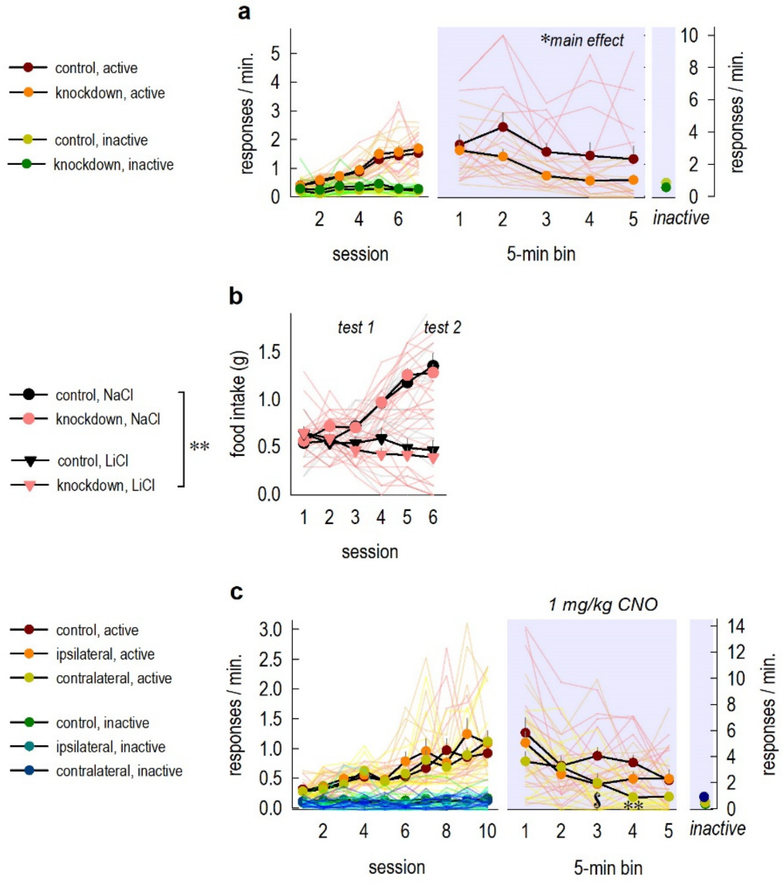

(a) Corresponds to main text figure 3b. (b) Corresponds to main text figure 3c. (d) Corresponds to main text figure 4g. Light lines represent individual mice; otherwise, text is the same as in the main text.

Suppl. fig.7. Uncropped gels corresponding to main text.

Allen et al, full gels
Main text. PSD95 at top, synaptophysin below

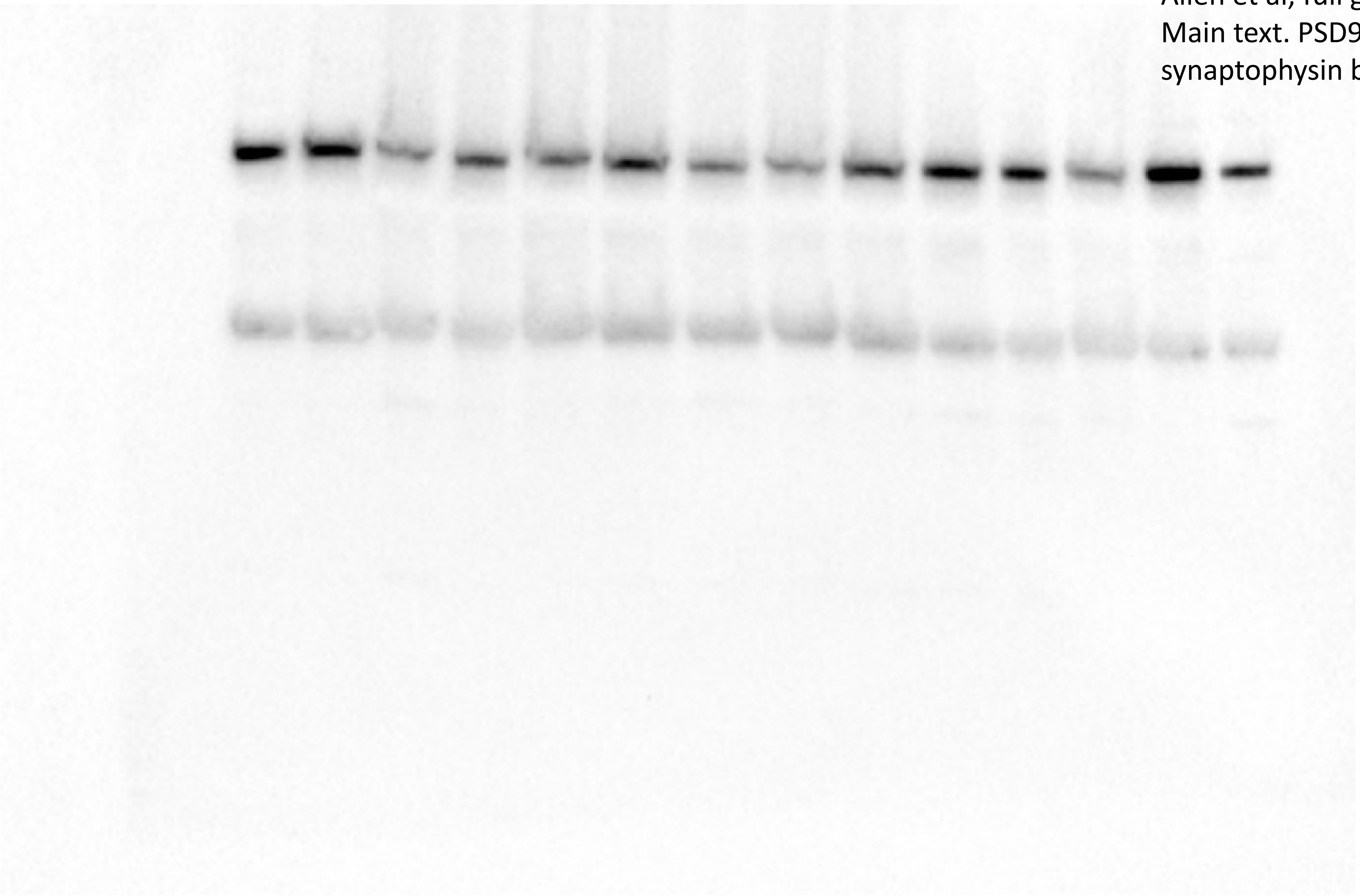

Suppl. fig.7. Uncropped gels corresponding to main text, continued.

Allen et al, full gels
Main text. Synaptophysin at middle as measured (over-exposed PSD95 at top)

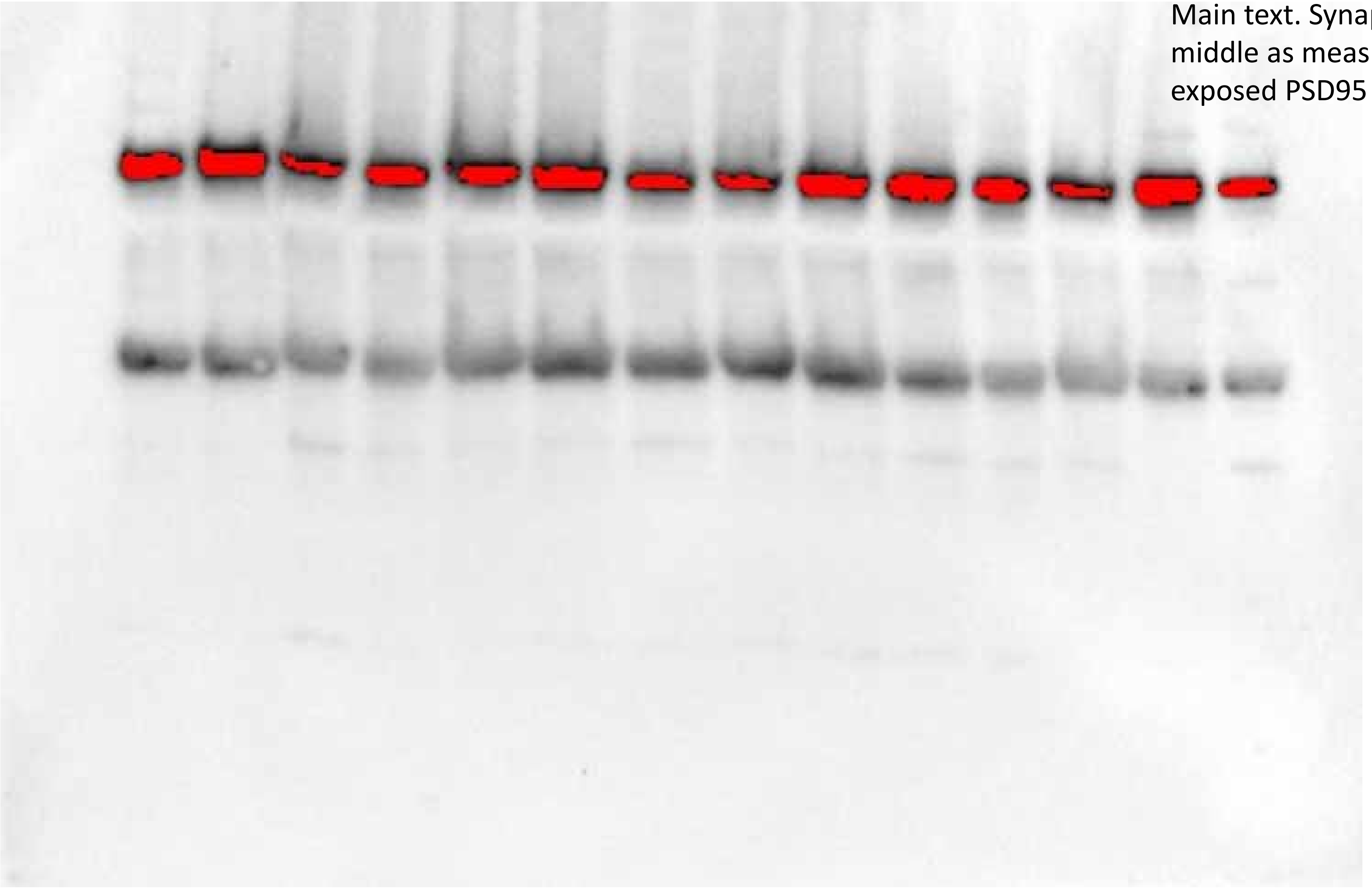

Suppl. fig.7. Uncropped gels corresponding to main text, continued.

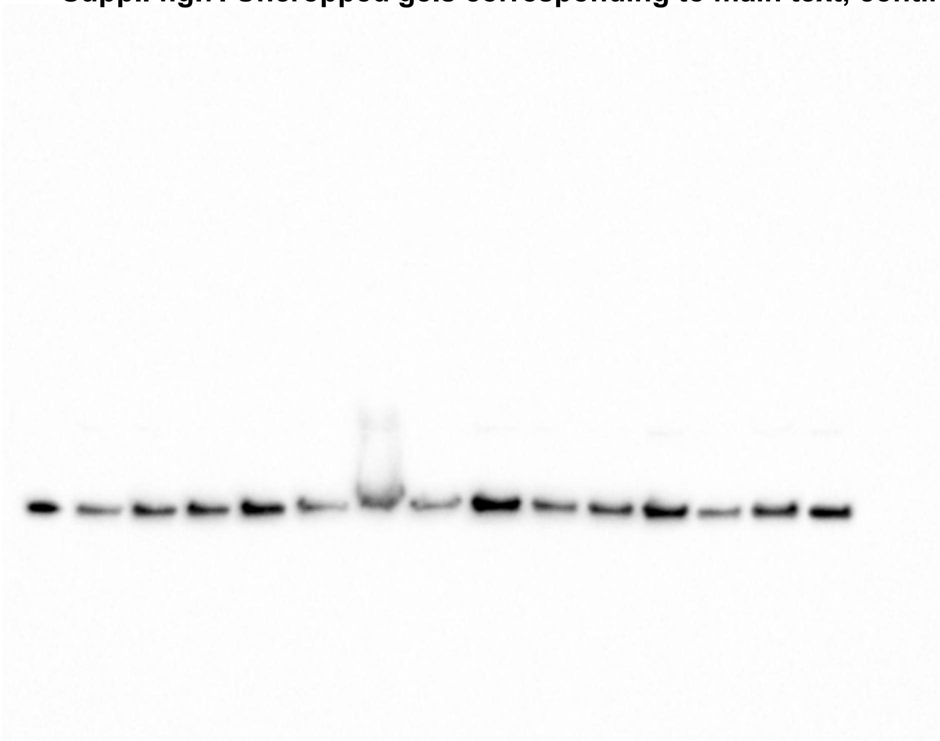

Allen et al, full gels
Main text. CNPase

Suppl. fig.7. Uncropped gels corresponding to main text, continued.

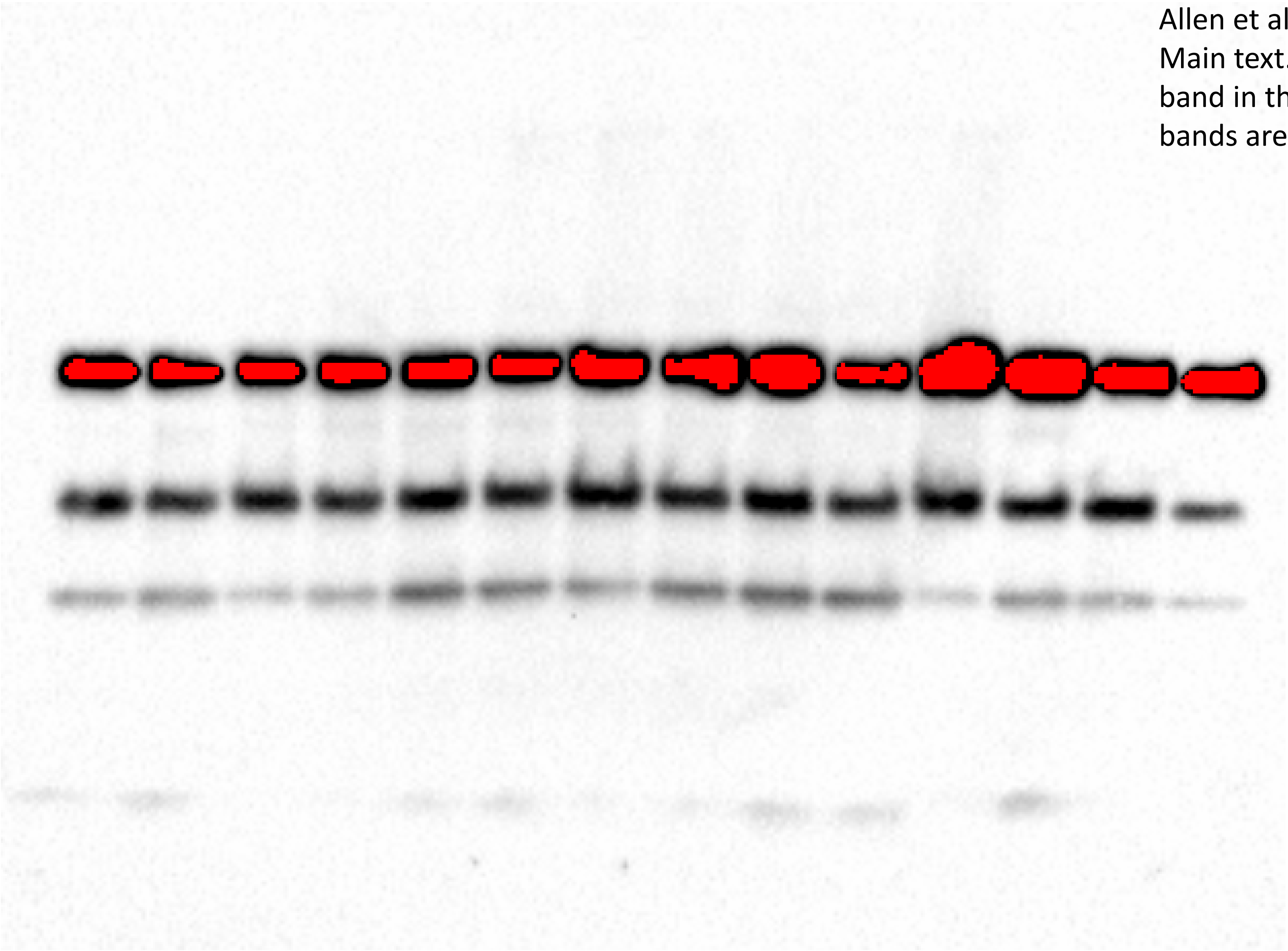

Allen et al, full gels
Main text. MC4R is the dark band in the middle. Other bands are other proteins.

Suppl. fig.7. Uncropped gels corresponding to main text, continued.

Allen et al, full gels
Rep. Hsp-70 loading control

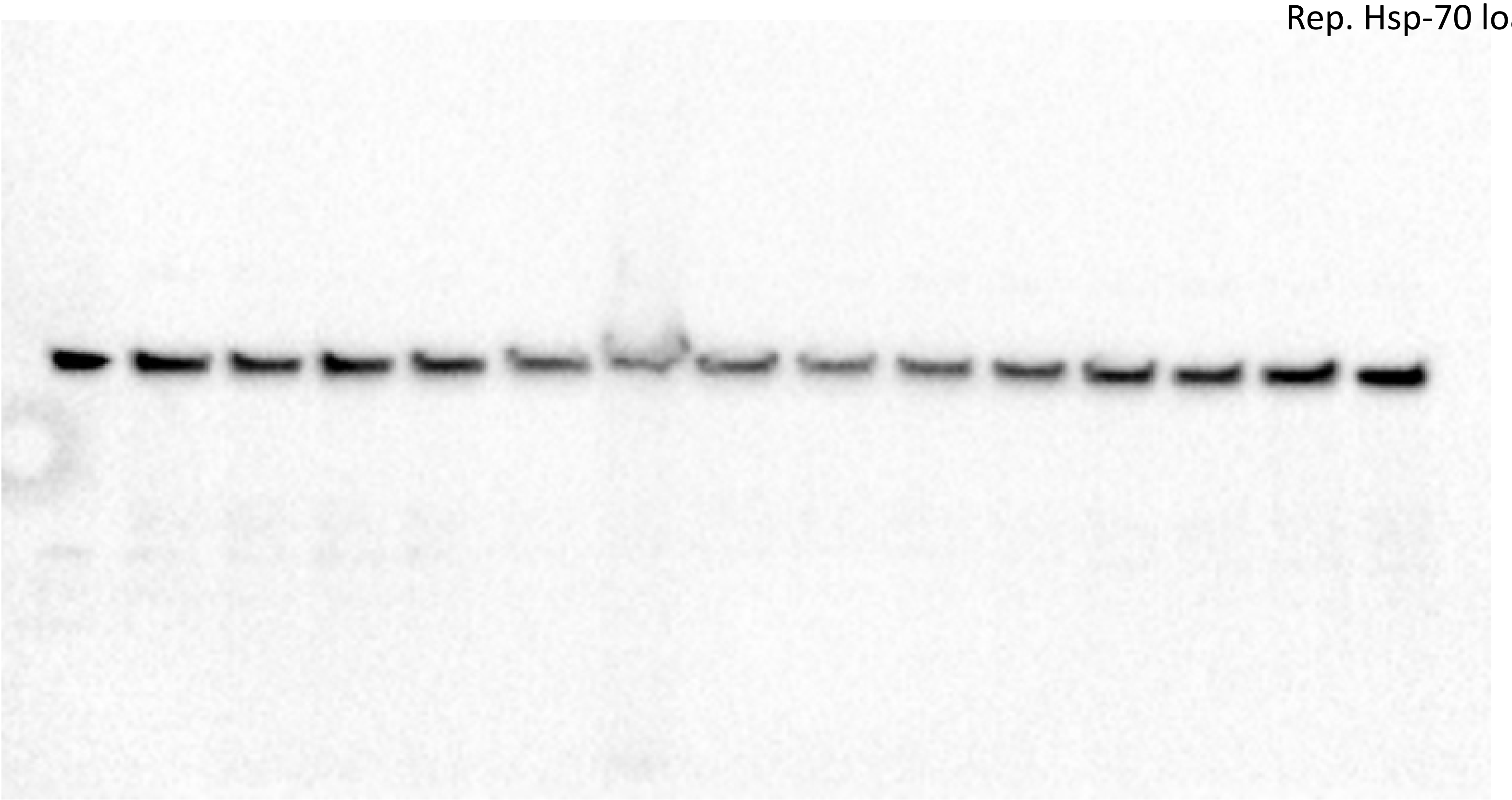

Suppl. fig.7. Uncropped gels corresponding to main text, continued.

Allen et al, full gels

Supplement follows

Suppl. fig.7. Uncropped gels corresponding to main text, continued.

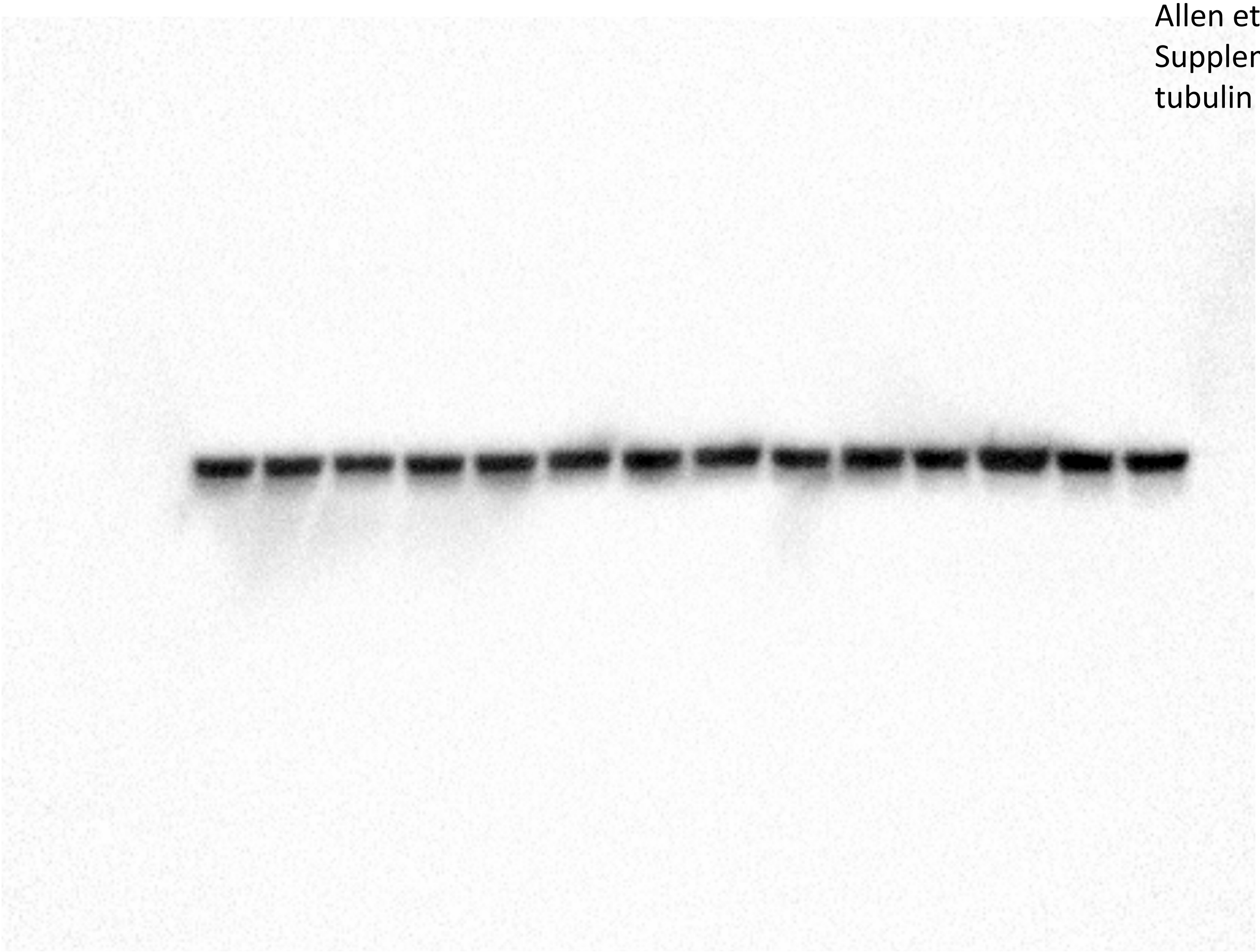

Allen et al, full gels Supplement. DMS alpha-tubulin

Suppl. fig.7. Uncropped gels corresponding to main text, continued.

Allen et al, full gels
Corresponding total protein

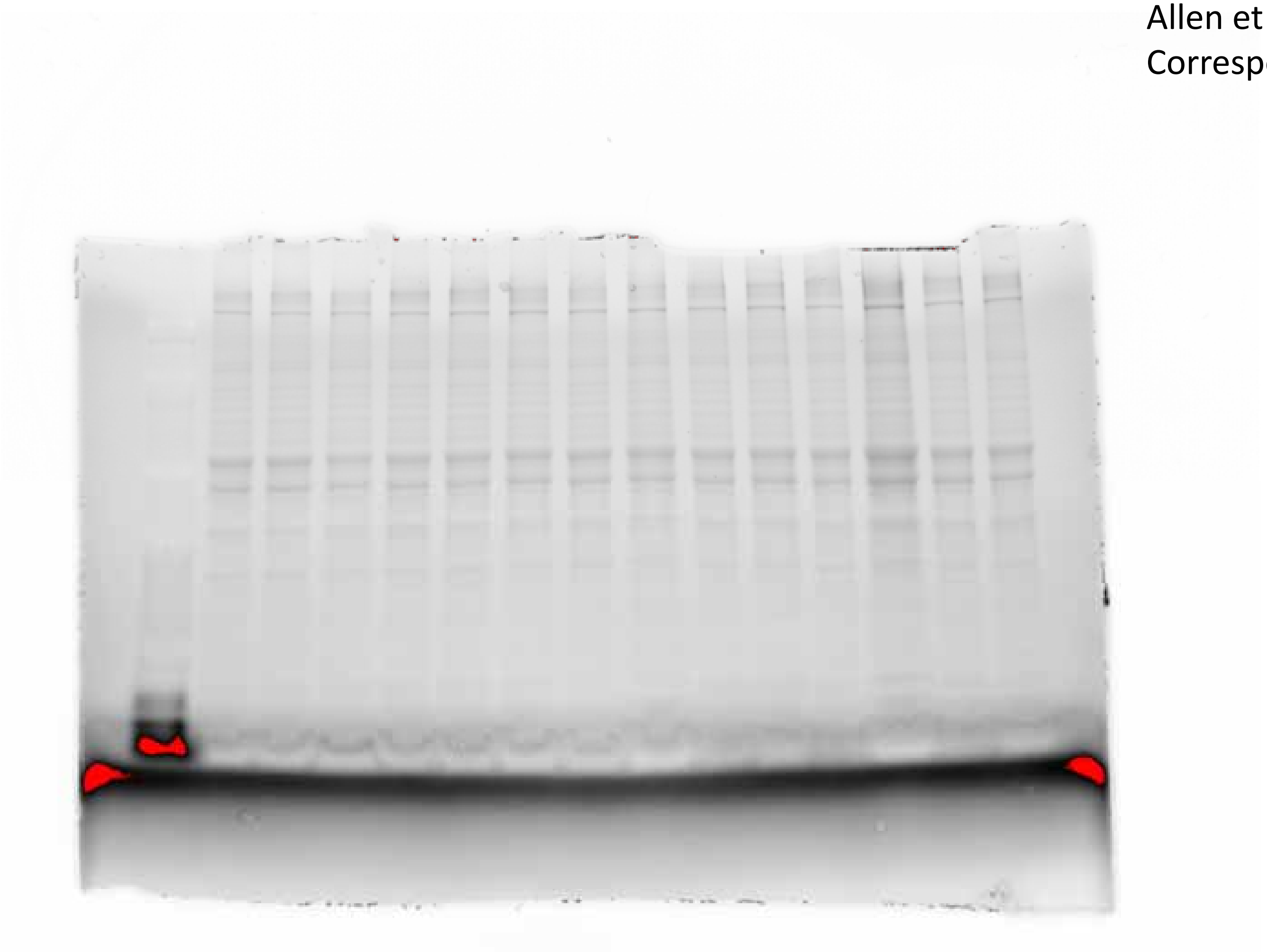

Suppl. fig.7. Uncropped gels corresponding to main text, continued.

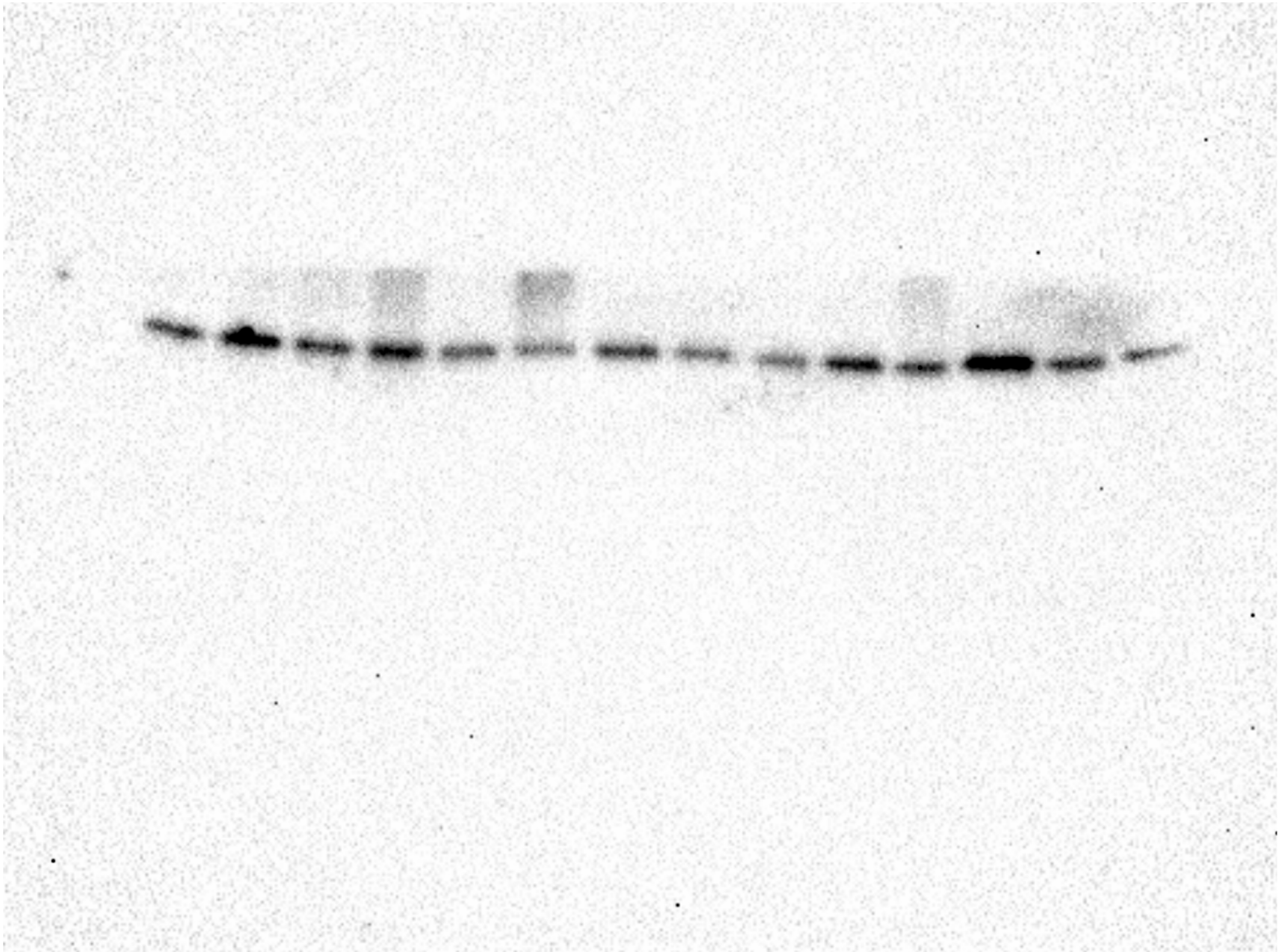

Allen et al, full gels Supplement. DMS calmodulin

Please note that we sometimes cut our membranes in order to incubate in different primary antibodies, so some gels will appear cut.

Suppl. fig.7. Uncropped gels corresponding to main text, continued.

Allen et al, full gels
Corresponding total protein

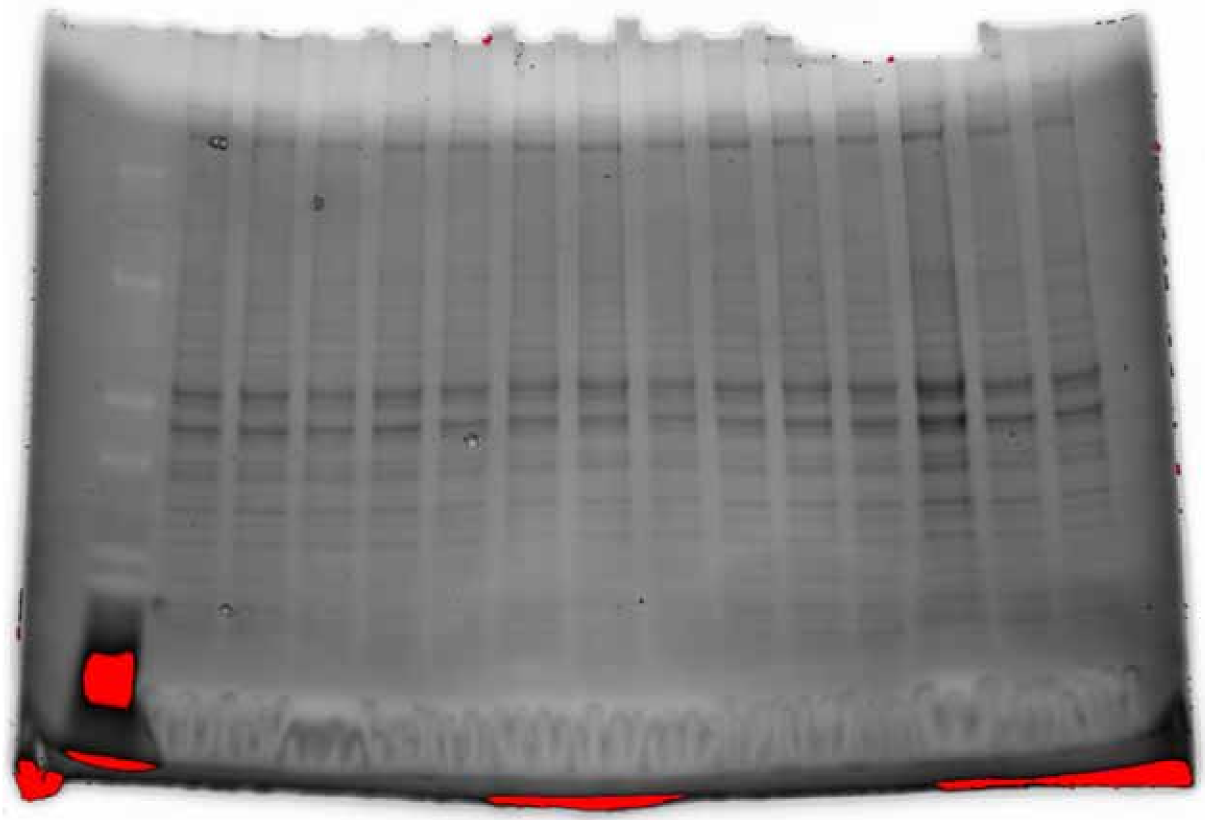

Suppl. fig.7. Uncropped gels corresponding to main text, continued.

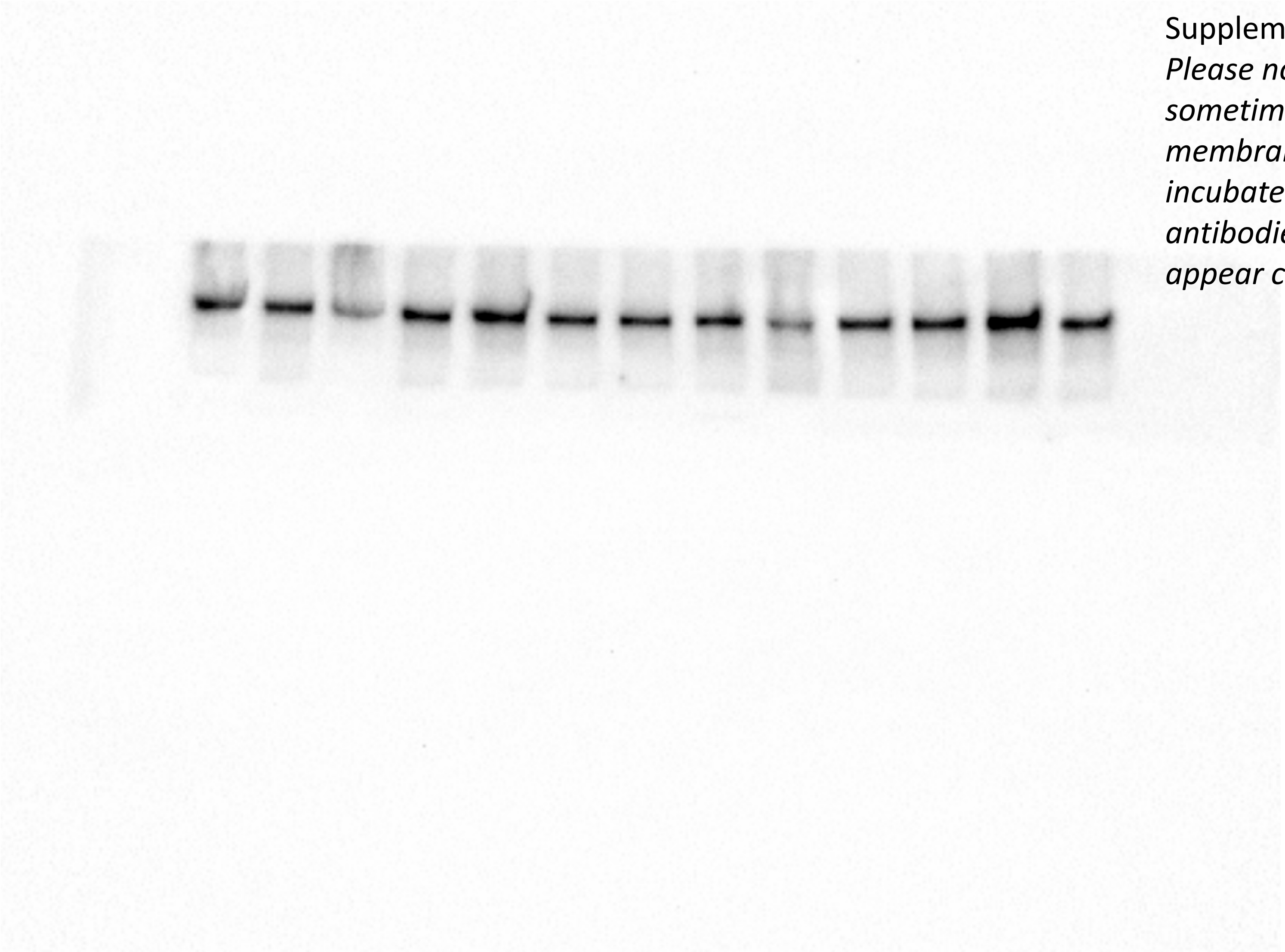

Allen et al, full gels Supplement. DMS GluN2B
Please note that we sometimes cut our membranes in order to incubate in different primary antibodies, so some gels will appear cut.

Suppl. fig.7. Uncropped gels corresponding to main text, continued.

Allen et al, full gels
Corresponding total protein

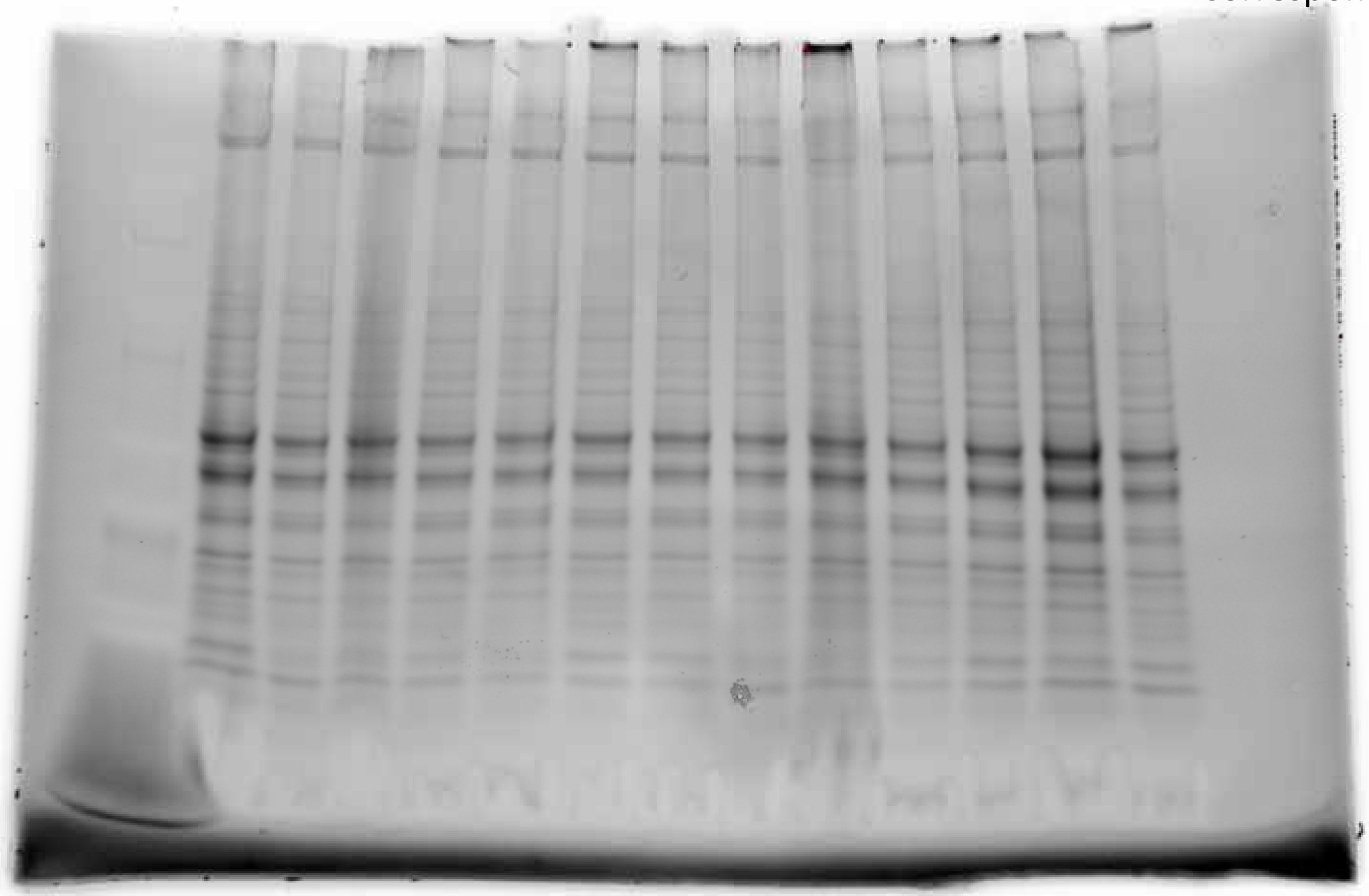

Suppl. fig.7. Uncropped gels corresponding to main text, continued.

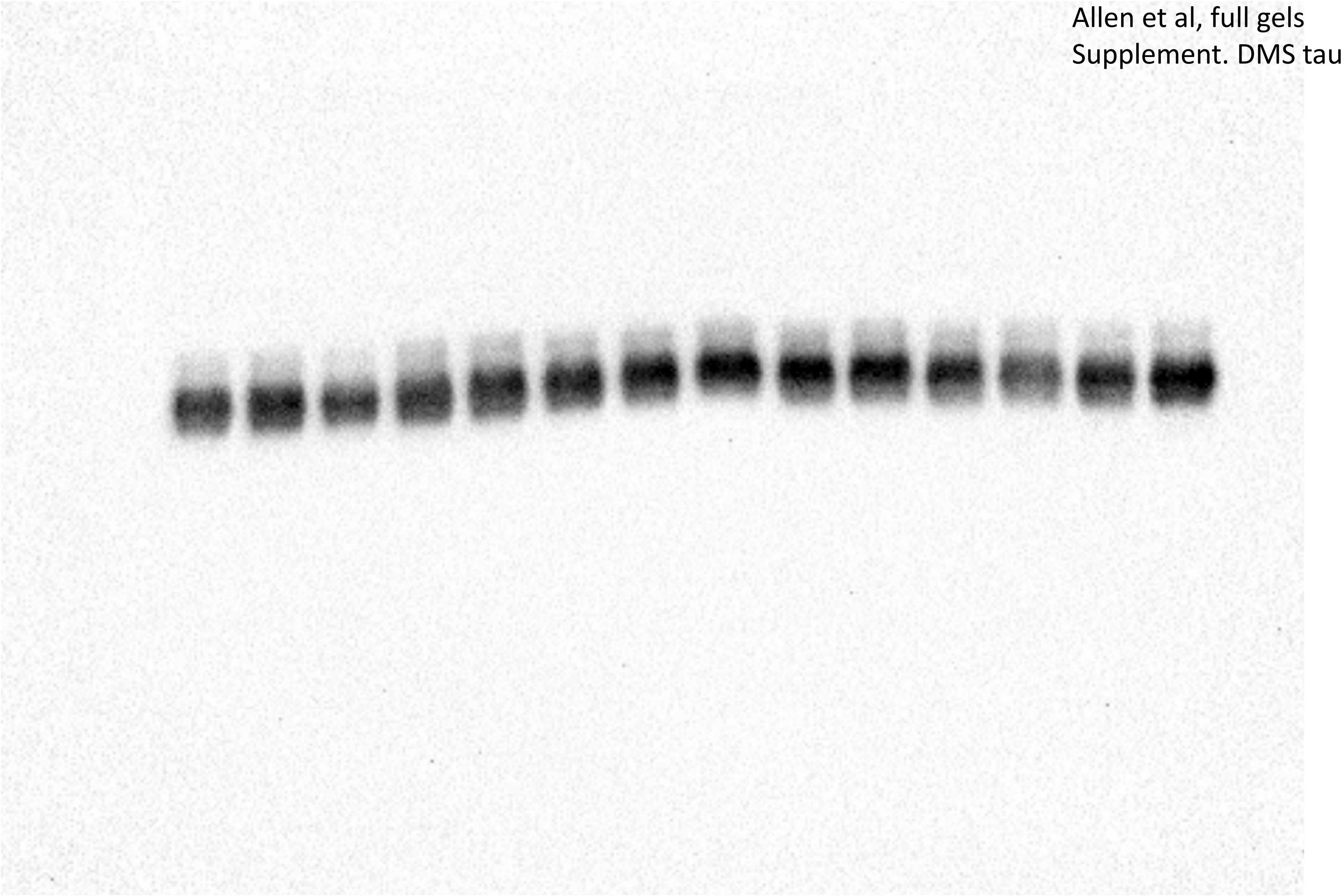

Suppl. fig.7. Uncropped gels corresponding to main text, continued.

Allen et al, full gels
Corresponding total protein

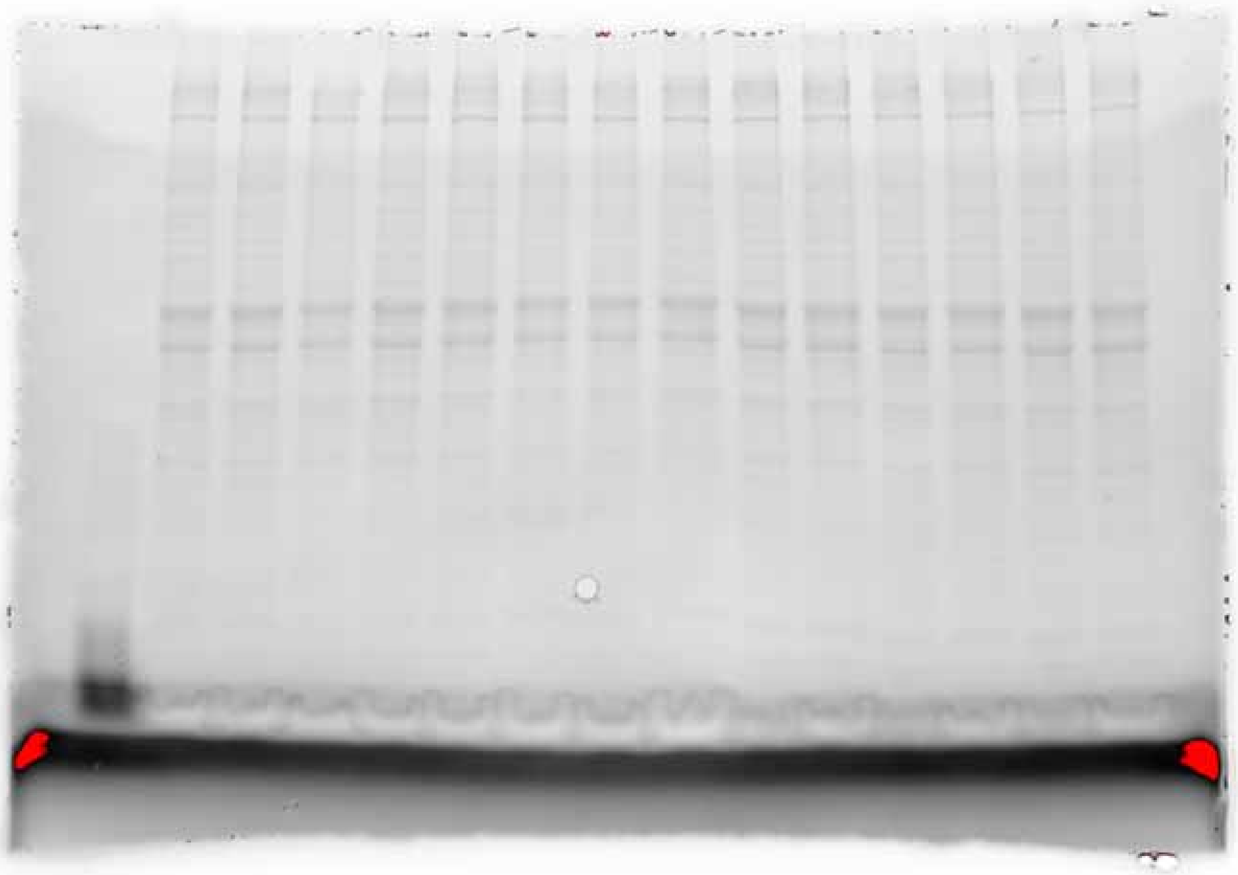

Suppl. fig.7. Uncropped gels corresponding to main text, continued.

Allen et al, full gels
Supplement. DMS TH

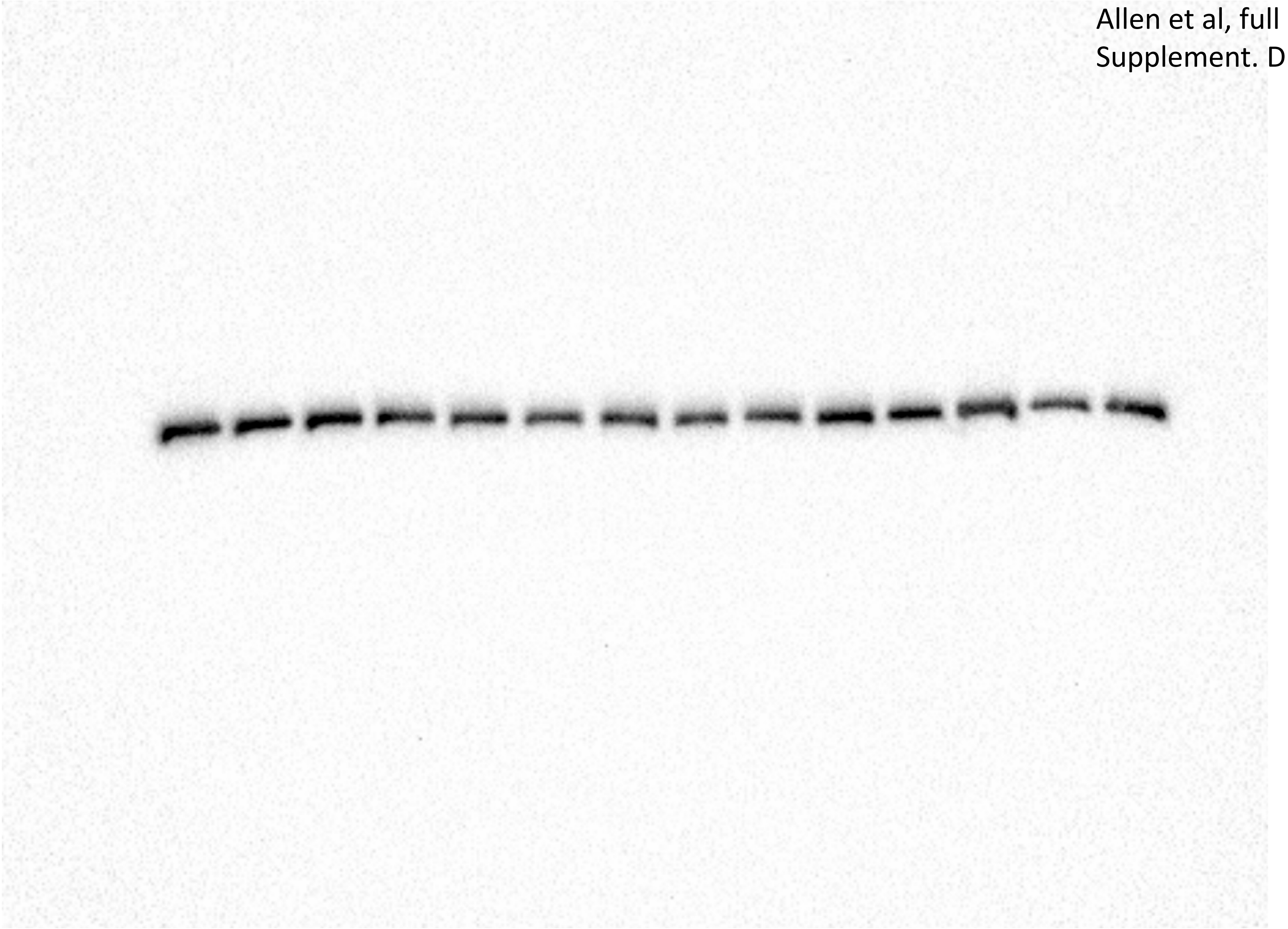

Suppl. fig.7. Uncropped gels corresponding to main text, continued.

Allen et al, full gels
Corresponding total protein

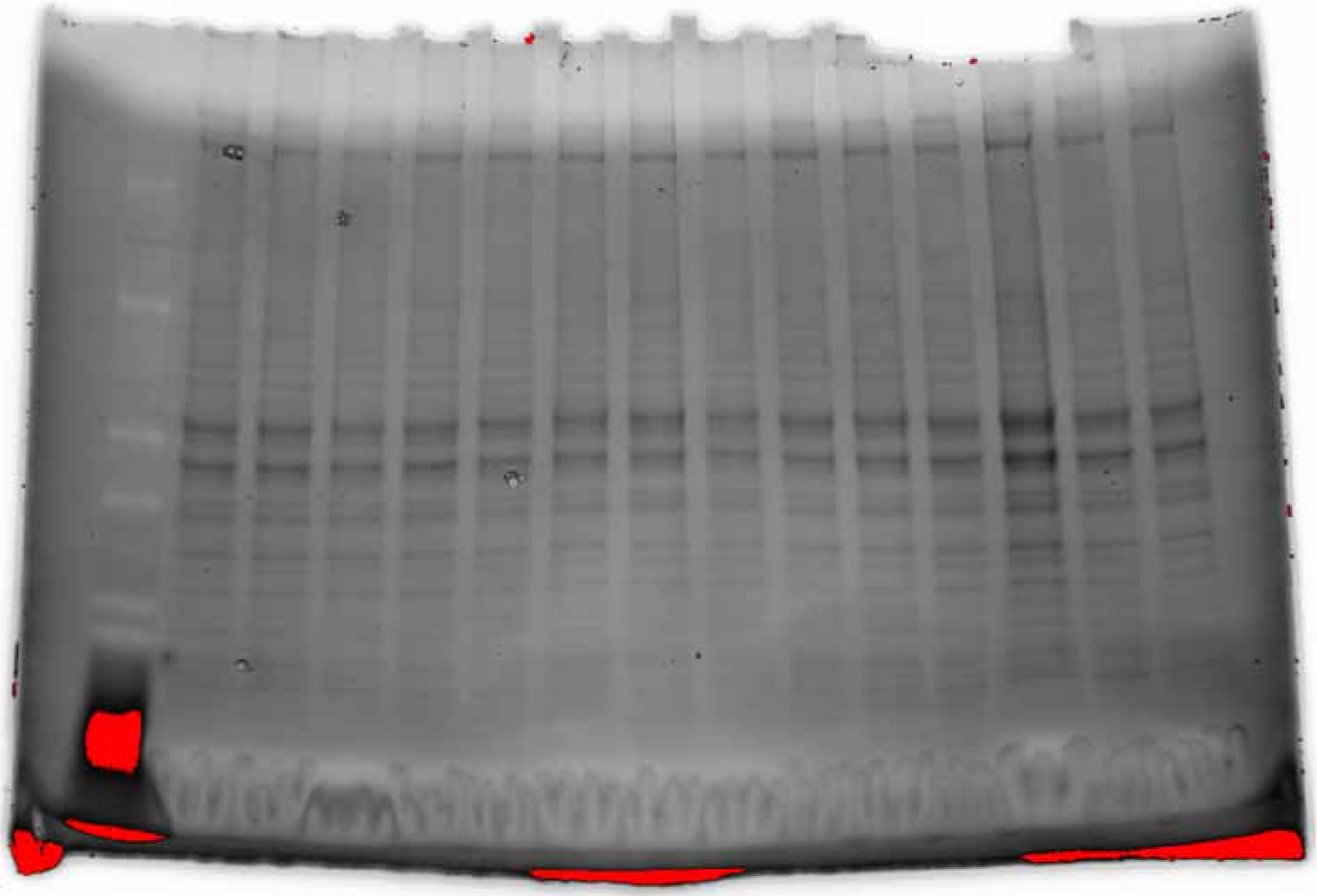

Suppl. fig.7. Uncropped gels corresponding to main text, continued.

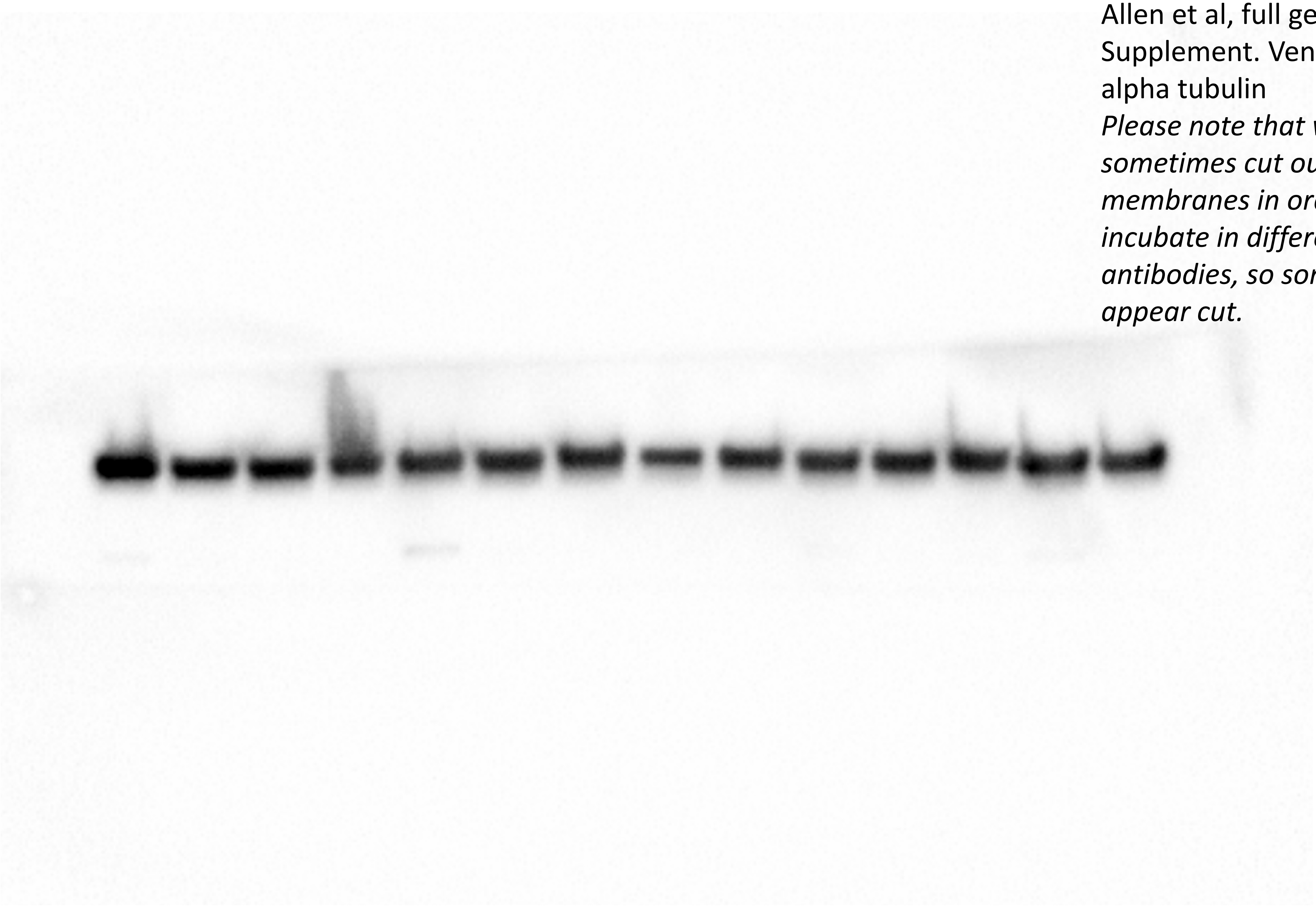

Allen et al, full gels
Supplement. Ventral striatum
alpha tubulin
Please note that we sometimes cut our membranes in order to incubate in different primary antibodies, so some gels will appear cut.

Suppl. fig.7. Uncropped gels corresponding to main text, continued.

Allen et al, full gels
Corresponding total protein

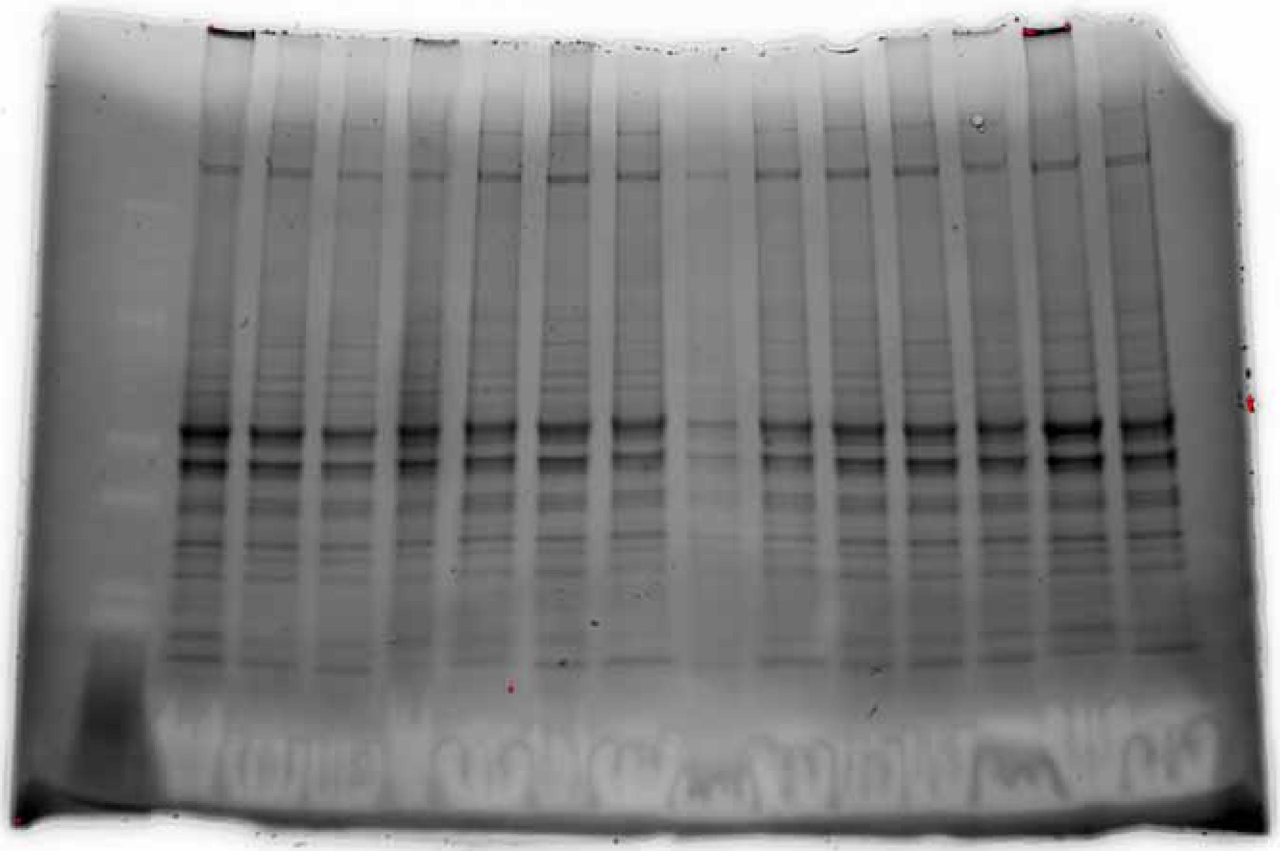

Suppl. fig.7. Uncropped gels corresponding to main text, continued.

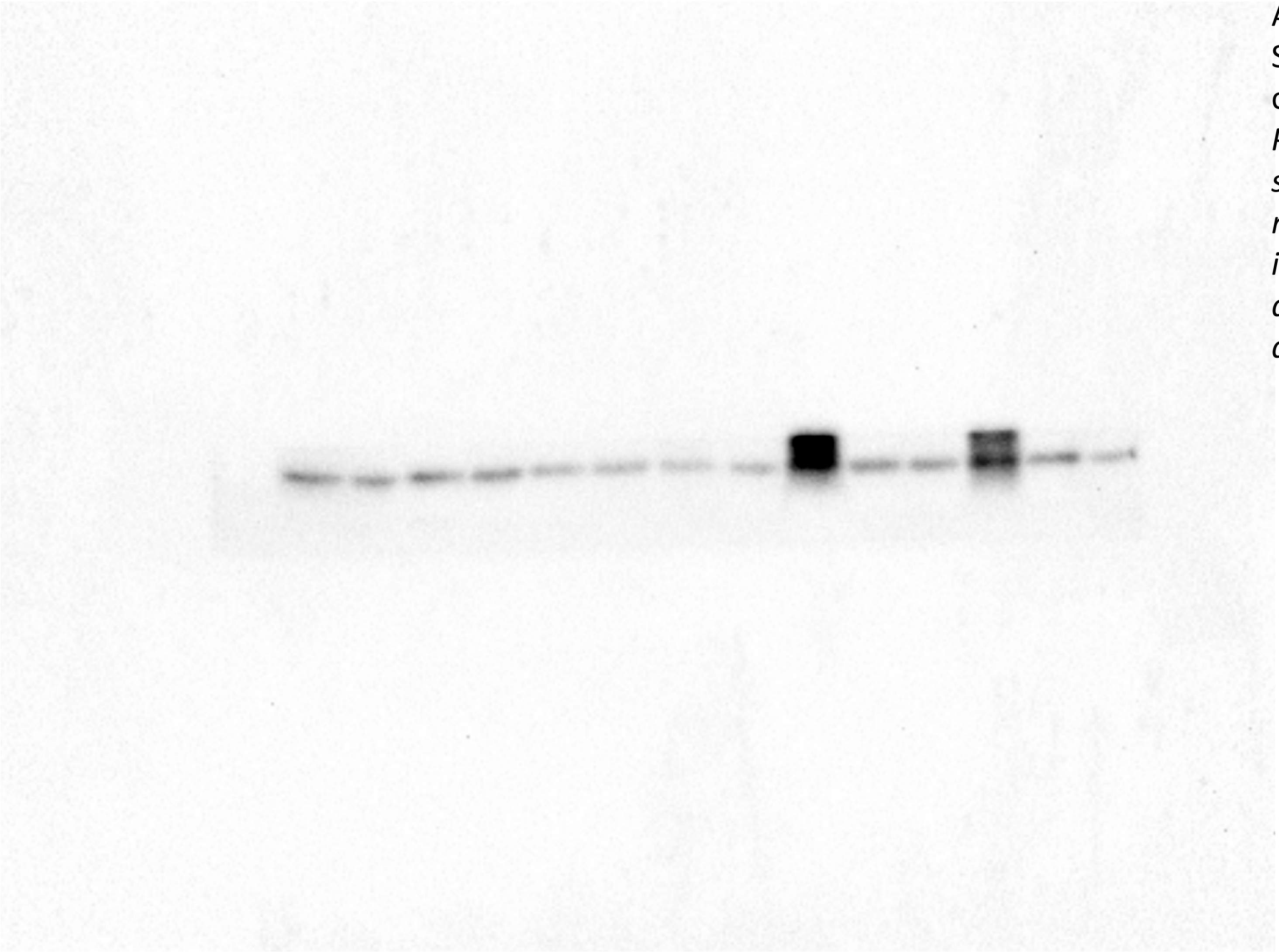

Allen et al, full gels
Supplement. Ventral striatum
calmodulin
Please note that we sometimes cut our membranes in order to incubate in different primary antibodies, so some gels will appear cut.

Suppl. fig.7. Uncropped gels corresponding to main text, continued.

Allen et al, full gels
Corresponding total protein

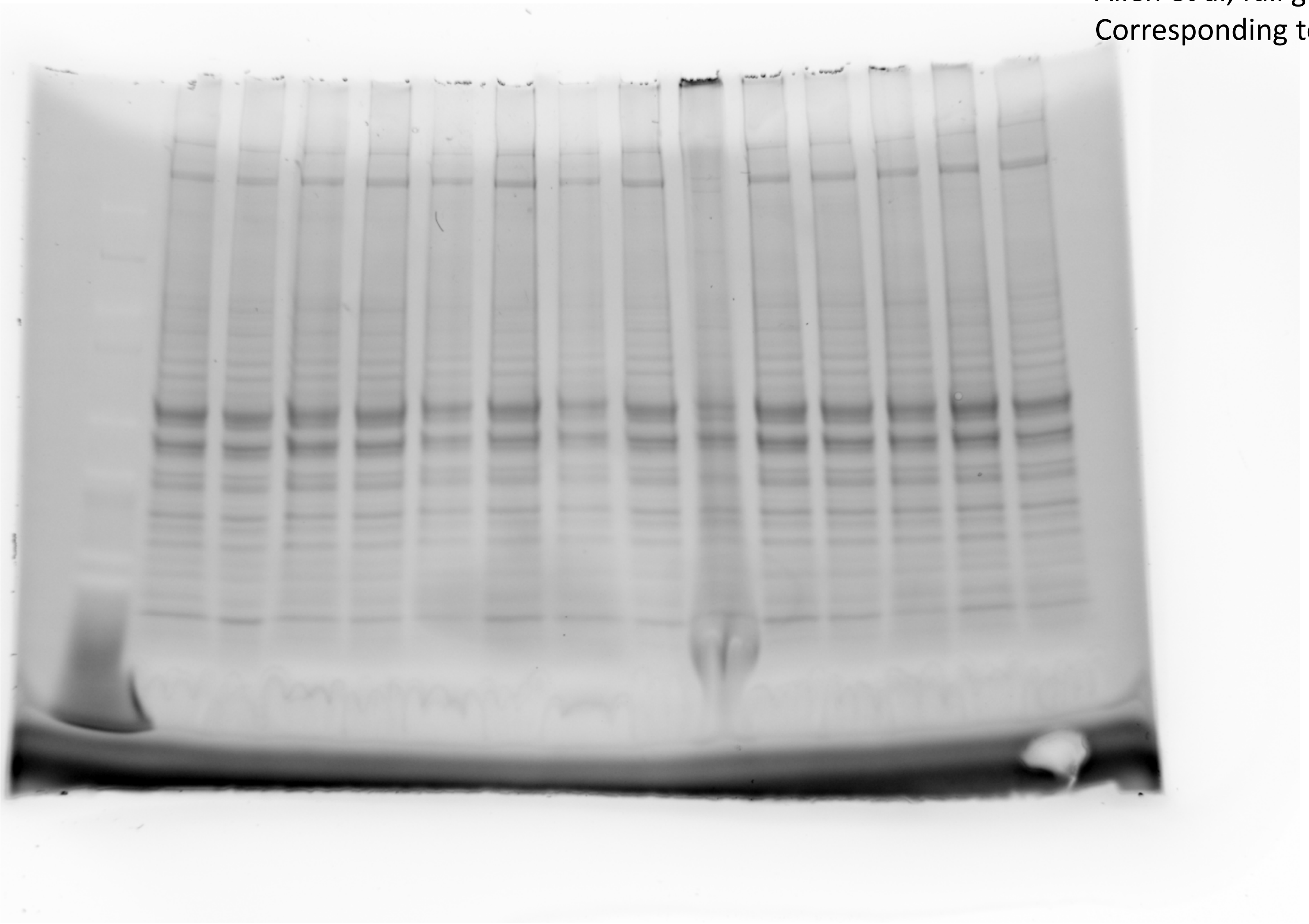

Suppl. fig.7. Uncropped gels corresponding to main text, continued.

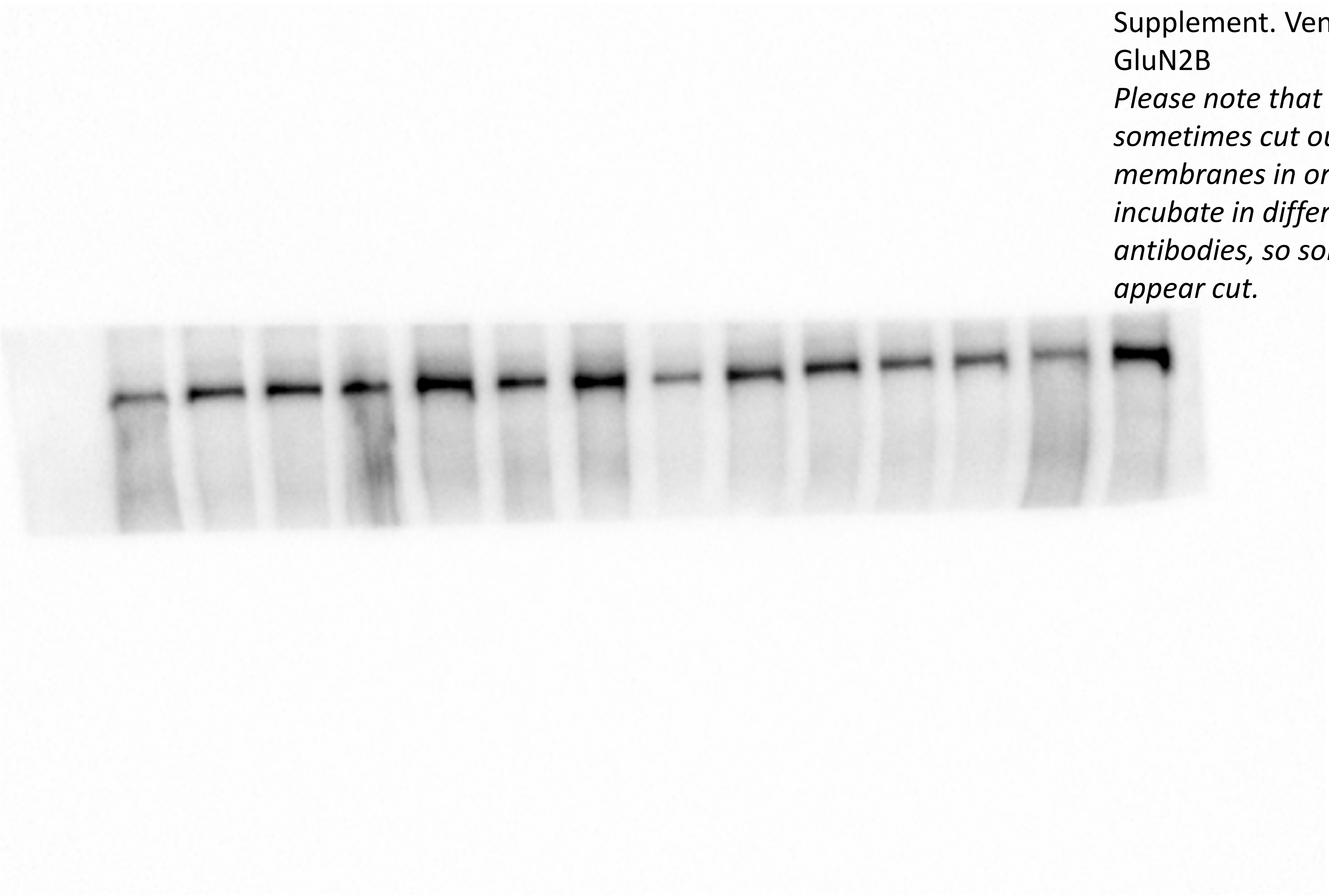

Allen et al, full gels
Supplement. Ventral striatum
GluN2B
Please note that we sometimes cut our membranes in order to incubate in different primary antibodies, so some gels will appear cut.

Suppl. fig.7. Uncropped gels corresponding to main text, continued.

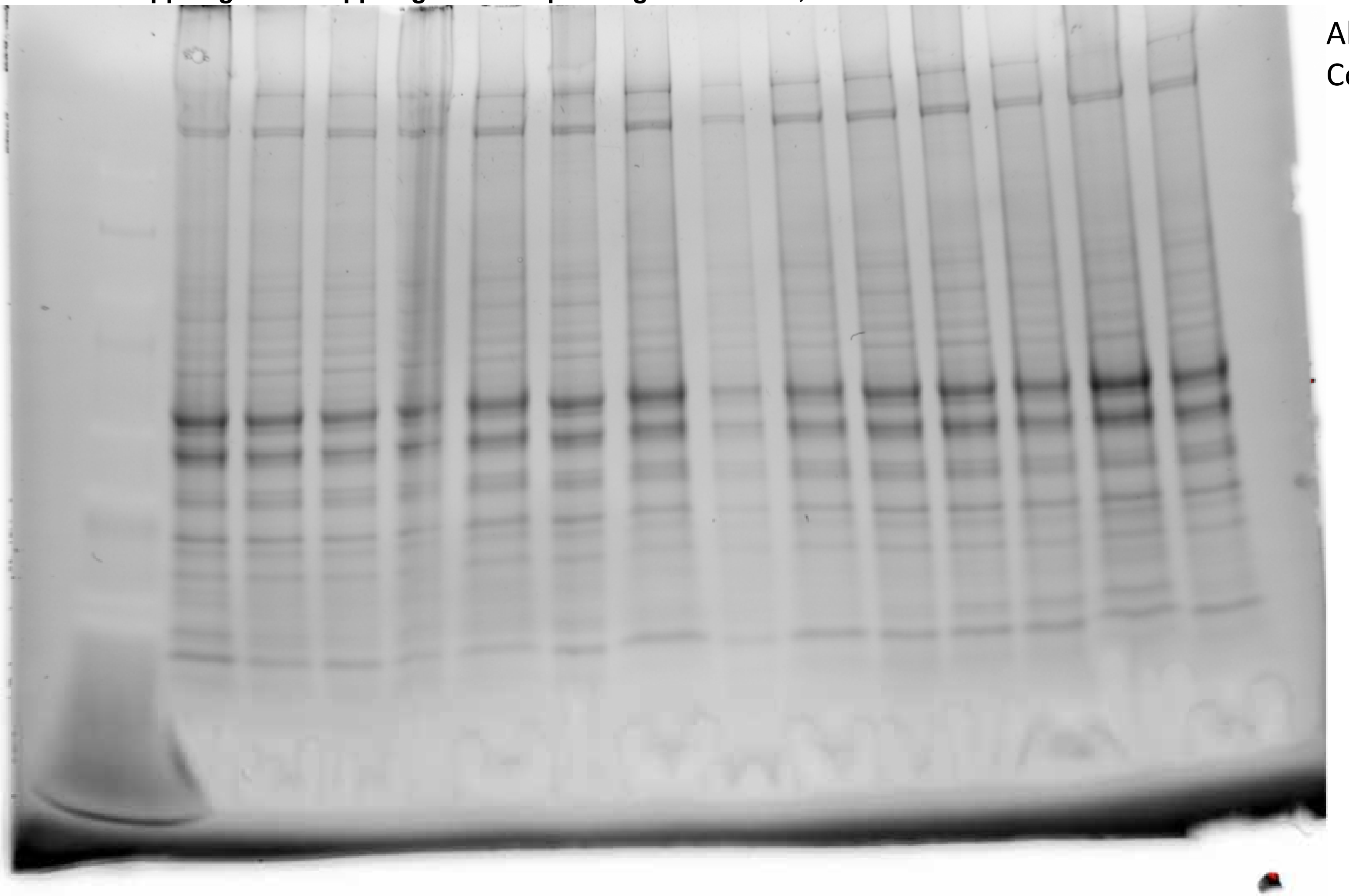

Allen et al, full gels
Corresponding total protein

Suppl. fig.7. Uncropped gels corresponding to main text, continued.

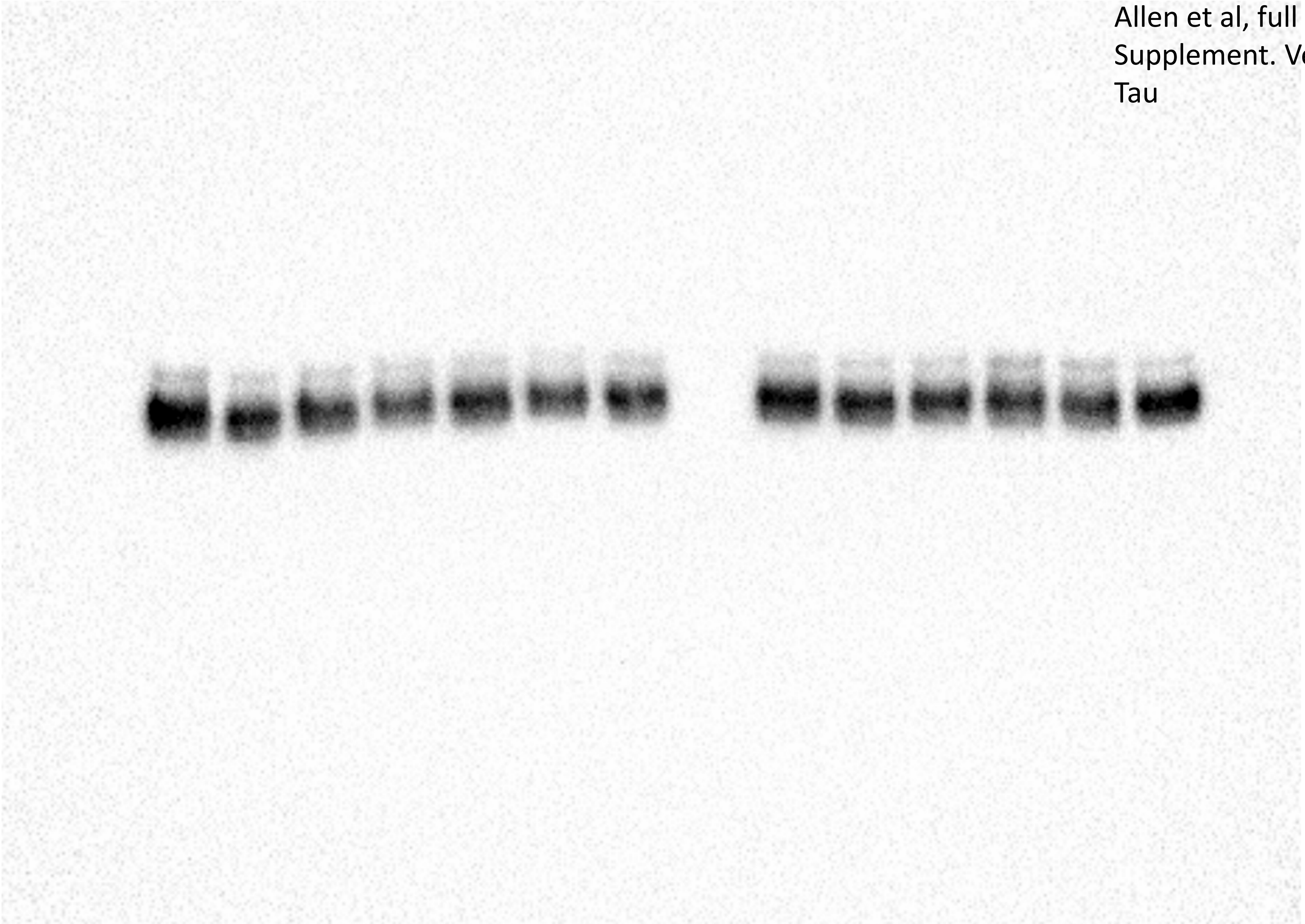

Suppl. fig.7. Uncropped gels corresponding to main text, continued.

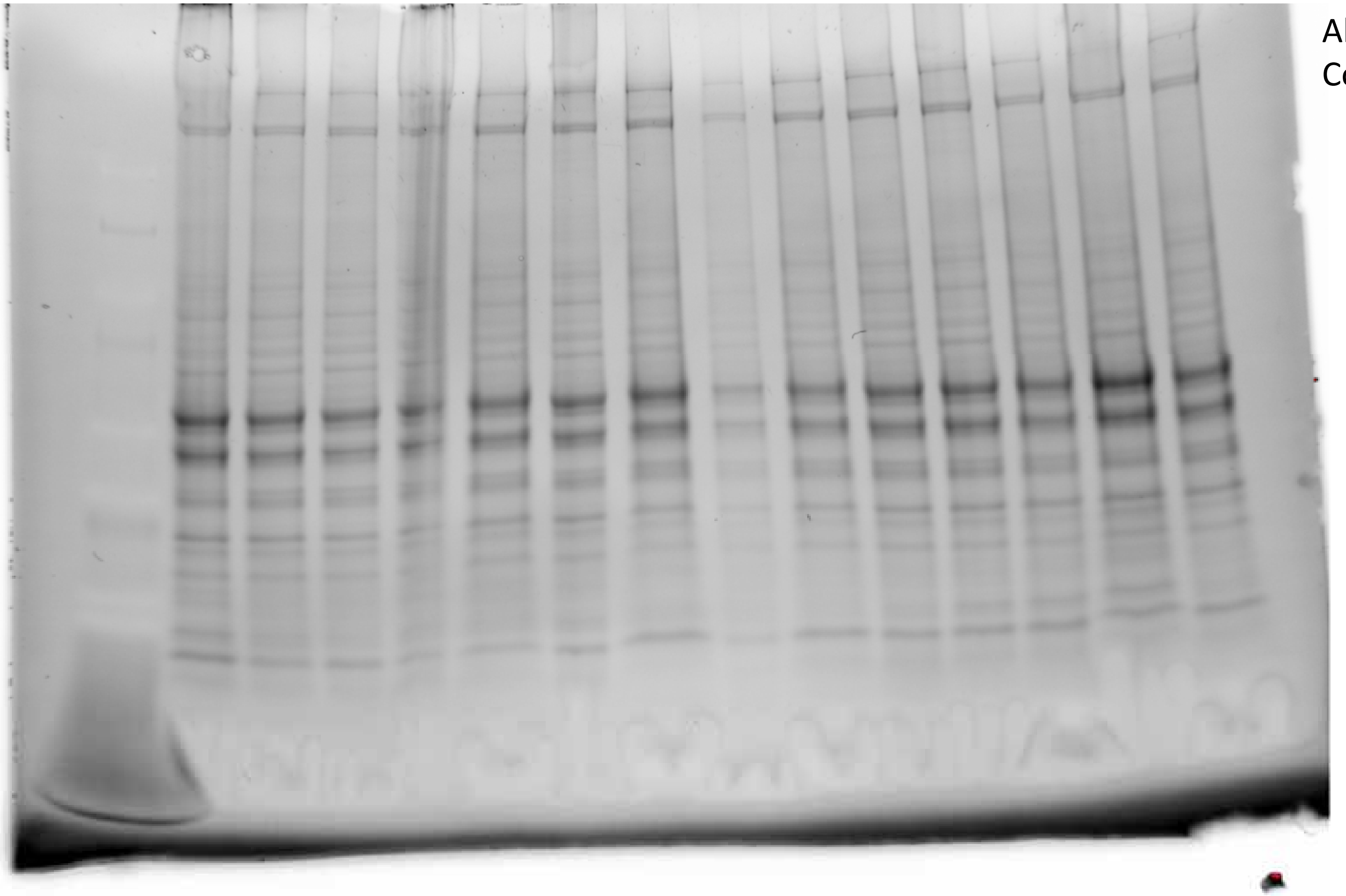

Allen et al, full gels
Corresponding total protein

Suppl. fig.7. Uncropped gels corresponding to main text, continued.

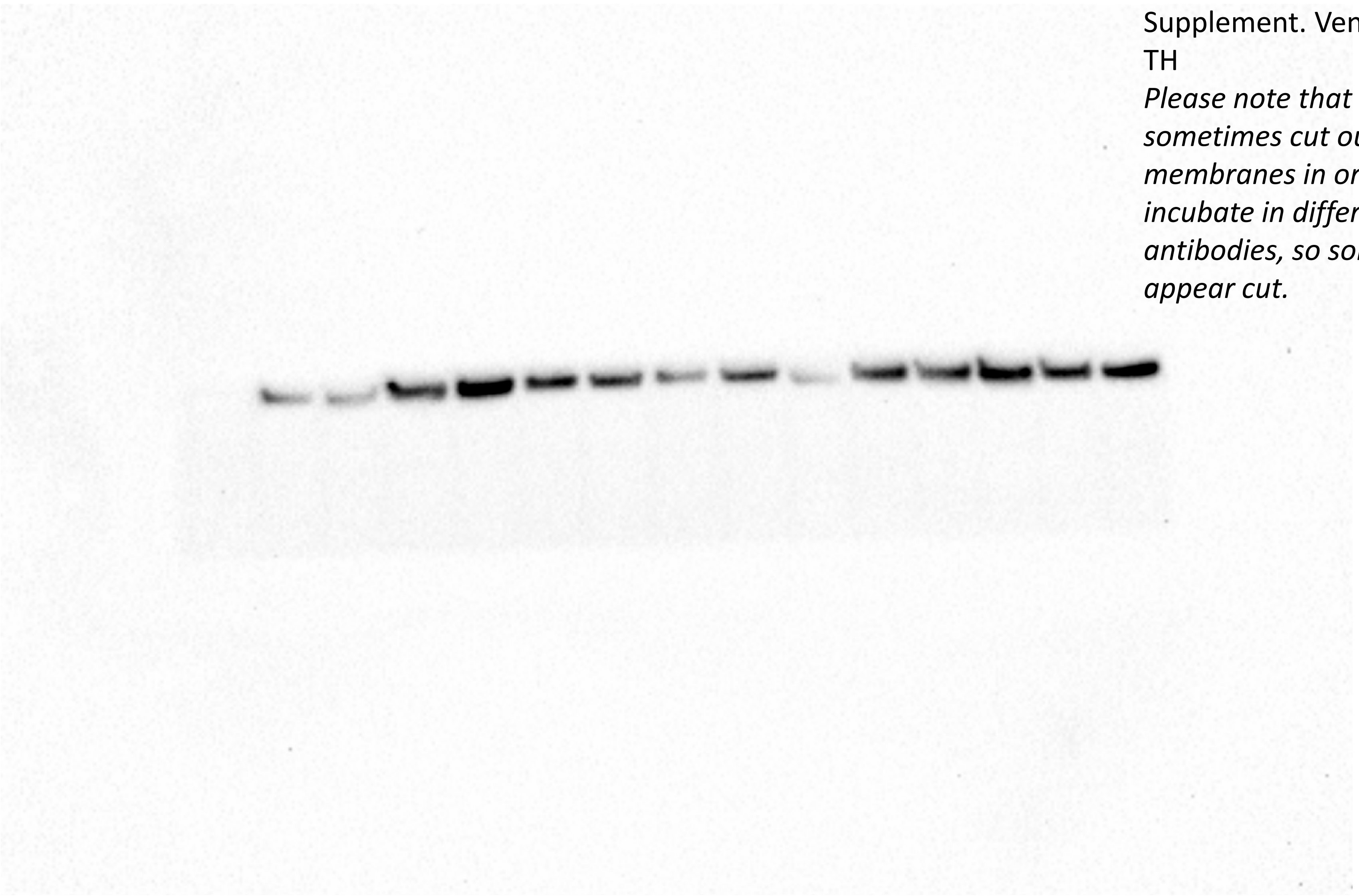

Allen et al, full gels
Supplement. Ventral striatum
TH
Please note that we sometimes cut our membranes in order to incubate in different primary antibodies, so some gels will appear cut.

Suppl. fig.7. Uncropped gels corresponding to main text, continued.

Allen et al, full gels
Corresponding total protein

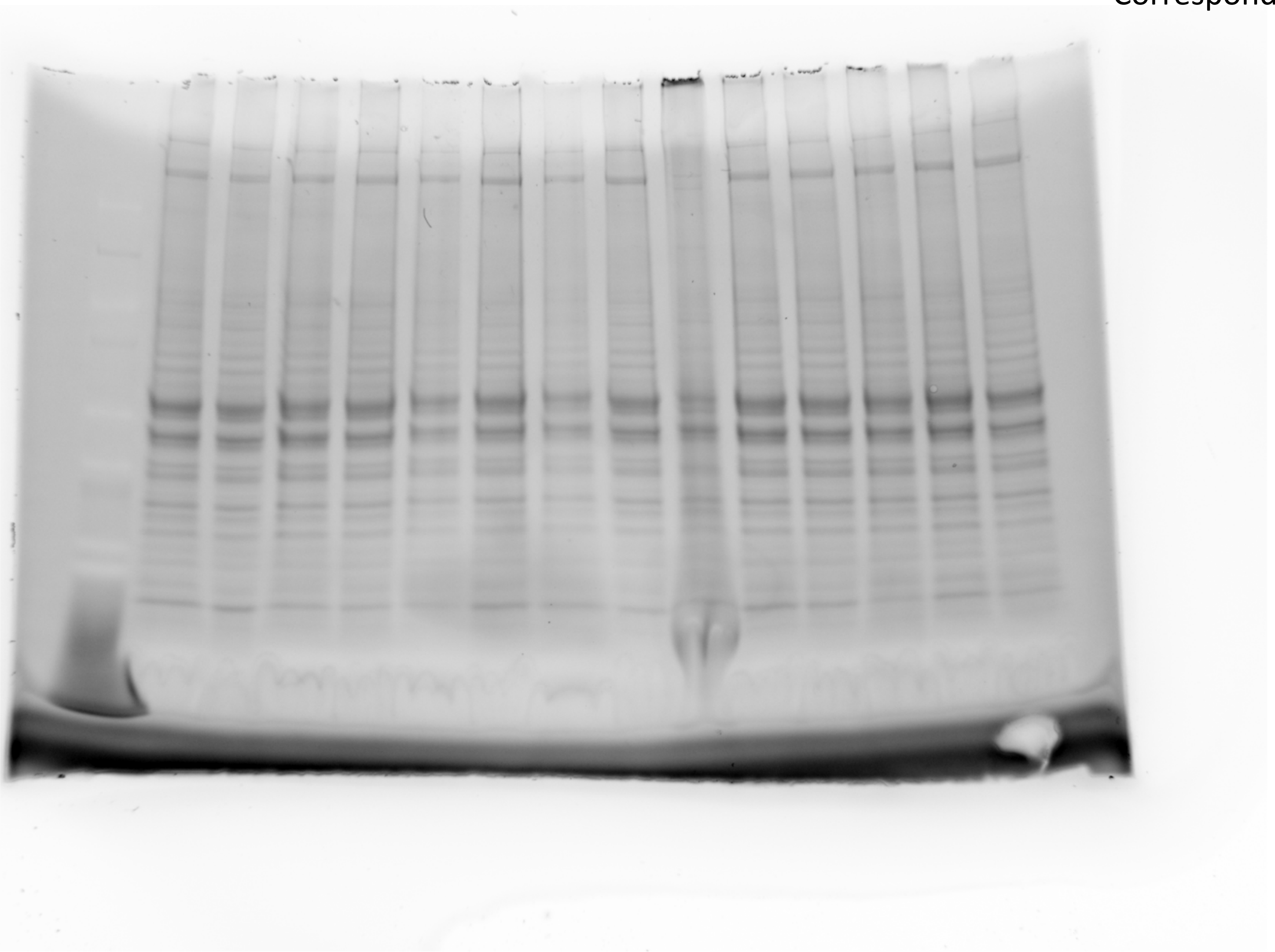

Supplementary References

[1] Rosen GD, Williams AG, Capra JA, Connolly MT, Cruz B, Lu L, Airey DC, Kulkarn IK, Williams RW (2000) The Mouse Brain Library @ www.mbl.org. Int Mouse Genome Conference 14(166).

[2] Van der Veen R, Abrous DN, de Kloet ER, Piazza PV, Koehl M (2008) Impact of intra- and interstrain cross-fostering on mouse maternal care. Genes Brain Behav 7:184-192.

[3] Heath CJ, Horst NK, Picciotto MR (2011) Oral nicotine consumption does not affect maternal care or early development in mice but results in modest hyperactivity in adolescence. Physiol Behav 101:764-769.
