# Supplementary material for: Inter-individual variability amplified through breeding reveals control of reward-related action strategies by Melanocortin-4 Receptor in the dorsomedial striatum
Source: Commun Biol. 2022 Feb 8;5:116. doi: 10.1038/s42003-022-03043-2 (PMC8825839; doi:10.1038/s42003-022-03043-2)
Supplement: Supplementary file 2 — Description of Additional Supplementary Files [file 42003_2022_3043_MOESM2_ESM.pdf]

Description of Additional Supplementary Files

File name: Supplementary Data 1.

Description: Raw data for main and supplementary figures.
