# Supplementary material for: Inter-individual variability amplified through breeding reveals control of reward-related action strategies by Melanocortin-4 Receptor in the dorsomedial striatum
Source: Commun Biol. 2022 Feb 8;5:116. doi: 10.1038/s42003-022-03043-2 (PMC8825839; doi:10.1038/s42003-022-03043-2)
Supplement: Supplementary file 4 — Reporting Summary [file 42003_2022_3043_MOESM4_ESM.pdf]

Corresponding author(s): Shannon Gourley
Last updated by author(s): Nov 24, 2021

Reporting Summary

Nature Portfolio wishes to improve the reproducibility of the work that we publish. This form provides structure for consistency and transparency in reporting. For further information on Nature Portfolio policies, see our Editorial Policies and the Editorial Policy Checklist.

Statistics

For all statistical analyses, confirm that the following items are present in the figure legend, table legend, main text, or Methods section.

n/a	Confirmed	
☐	☒	The exact sample size (*n*) for each experimental group/condition, given as a discrete number and unit of measurement
☐	☒	A statement on whether measurements were taken from distinct samples or whether the same sample was measured repeatedly
☐	☒	The statistical test(s) used AND whether they are one- or two-sided<br>*Only common tests should be described solely by name; describe more complex techniques in the Methods section.*
☐	☒	A description of all covariates tested
☐	☒	A description of any assumptions or corrections, such as tests of normality and adjustment for multiple comparisons
☐	☒	A full description of the statistical parameters including central tendency (e.g. means) or other basic estimates (e.g. regression coefficient) AND variation (e.g. standard deviation) or associated estimates of uncertainty (e.g. confidence intervals)
☐	☒	For null hypothesis testing, the test statistic (e.g. *F*, *t*, *r*) with confidence intervals, effect sizes, degrees of freedom and *P* value noted<br>*Give P values as exact values whenever suitable.*
☒	☐	For Bayesian analysis, information on the choice of priors and Markov chain Monte Carlo settings
☒	☐	For hierarchical and complex designs, identification of the appropriate level for tests and full reporting of outcomes
☒	☐	Estimates of effect sizes (e.g. Cohen's *d*, Pearson's *r*), indicating how they were calculated

Our web collection on statistics for biologists contains articles on many of the points above.

Software and code

Policy information about availability of computer code

Data collection	no software used
Data analysis	SPSS v.28 and SigmaPlot v.11 and 14.5 were used to analyze data.

For manuscripts utilizing custom algorithms or software that are central to the research but not yet described in published literature, software must be made available to editors and reviewers. We strongly encourage code deposition in a community repository (e.g. GitHub). See the Nature Portfolio guidelines for submitting code & software for further information.

Data

Policy information about availability of data

All manuscripts must include a data availability statement. This statement should provide the following information, where applicable:
- Accession codes, unique identifiers, or web links for publicly available datasets
- A description of any restrictions on data availability
- For clinical datasets or third party data, please ensure that the statement adheres to our policy

Data are available in the supplementary materials.

Field-specific reporting

Life sciences study design

All studies must disclose on these points even when the disclosure is negative.

Sample size	Sample sizes were determined based on the behavioral phenotype of the mice -- i.e., to study individual differences -- or based on prior experiments.
Data exclusions	Exclusions: Values >2 standard deviations outside of the mean were considered outliers; thus, one mouse from each group in the "disconnection" experiment in the final figure generated multiple outlying values during training and was excluded. Any mice with misplaced viral vectors were also excluded. Finally, 1 mouse in the delay discounting procedure did not nose poke and was excluded.
Replication	Replication strategies for each experiment are detailed in the corresponding figure captions. Note that replications comprised independent cohorts of mice, not resampling of the same mice.
Randomization	allocation to groups is detailed in the breeding strategy section
Blinding	blinding was applied in imaging experiments, which is explicitly stated

Reporting for specific materials, systems and methods

We require information from authors about some types of materials, experimental systems and methods used in many studies. Here, indicate whether each material, system or method listed is relevant to your study. If you are not sure if a list item applies to your research, read the appropriate section before selecting a response.

Materials & experimental systems

n/a	Involved in the study
☐	☒ Antibodies
☒	☐ Eukaryotic cell lines
☒	☐ Palaeontology and archaeology
☐	☒ Animals and other organisms
☒	☐ Human research participants
☒	☐ Clinical data
☒	☐ Dual use research of concern

Methods

n/a	Involved in the study
☒	☐ ChIP-seq
☒	☐ Flow cytometry
☒	☐ MRI-based neuroimaging

Antibodies

Antibodies used	Primary antibodies were PSD-95 (Ms, Cell Signaling #3450, 1:1000), Synaptophysin (Rb, Abcam #32127, 1:20,000), CNPase [Ms, Millipore (multiple tested), 1:1000], MC4R (Rb, Abcam #150419, 1:1000), Tau (Rb, Cell Signaling #46687; 1:1000), Tyrosine hydroxylase (Rb, Sigma #AB152; 1:1000), GluN2B (Ms, Novus Biologicals #NB100-74475; 1:500), Alpha-tubulin (Rb, Cell Signaling #3873; 1:1000), Calmodulin (Rb, Cell Signaling #35944; 1:1000).
Validation	All antibodies are commercially available and were validated by the manufacturer; details for each antibody can be found via the identification information provided above.

Animals and other organisms

Policy information about studies involving animals; ARRIVE guidelines recommended for reporting animal research

Laboratory animals	Initial experiments bred mice with particular behavioral traits and tested their offspring. These mice were maintained on a C57BL/6 background and expressed Thy1-driven YFPH (Jackson Labs), allowing us to visualize neurons and enumerate dendritic spines in some experiments. In experiments in which we manipulated Mc4r, mice were homozygous for a 'floxed' Mc4r gene (Jackson Labs). These mice were maintained on a mixed C57BL/6J-129S1/SvImJ background. Mice were weaned from the dam at or soon after postnatal day (P) 21 and housed in single-sex cages with siblings or unrelated mice of the same age. Mice were maintained on a 12-hour light cycle (0700 on) and provided food and water ad libitum except during food-reinforced behavioral testing when food was restricted to motivate responding. Experiments used both sexes.
Wild animals	*Provide details on animals observed in or captured in the field; report species, sex and age where possible. Describe how animals were caught and transported and what happened to captive animals after the study (if killed, explain why and describe method; if released, say where and when) OR state that the study did not involve wild animals.*
Field-collected samples	*For laboratory work with field-collected samples, describe all relevant parameters such as housing, maintenance, temperature, photoperiod and end-of-experiment protocol OR state that the study did not involve samples collected from the field.*
Ethics oversight	Procedures were approved by the Emory University IACUC.

Note that full information on the approval of the study protocol must also be provided in the manuscript.
